# Seropositivity and geographical distribution of *Strongyloides stercoralis* in Australia: A study of pathology laboratory data from 2012–2016

**DOI:** 10.1371/journal.pntd.0009160

**Published:** 2021-03-09

**Authors:** Jennifer Shield, Sabine Braat, Matthew Watts, Gemma Robertson, Miles Beaman, James McLeod, Robert W. Baird, Julie Hart, Jennifer Robson, Rogan Lee, Stuart McKessar, Suellen Nicholson, Johanna Mayer-Coverdale, Beverley-Ann Biggs

**Affiliations:** 1 Departments of Medicine and Infectious Diseases at the Doherty Institute, University of Melbourne, Melbourne, Victoria, Australia; 2 Department of Pharmacy and Applied Science, La Trobe University, Bendigo, Victoria, Australia; 3 Melbourne School of Population and Global Health, University of Melbourne, Parkville, Victoria, Australia; 4 Centre for Infectious Diseases and Microbiology, Pathology West-ICPMR and Marie Bashir Institute, University of Sydney, Westmead Hospital, Westmead, New South Wales, Australia; 5 Melbourne Pathology, Collingwood, Victoria, Australia; 6 PathWest Laboratory Medicine, Nedlands, Western Australia, Australia; 7 Territory Pathology, Alice Springs Hospital, Alice Springs, Northern Territory, Australia; 8 Territory Pathology, Royal Darwin Hospital, Tiwi, Northern Territory, Australia; 9 Sullivan Nicolaides Pathology, Bowen Hills, Queensland, Australia; 10 SA Pathology, Adelaide, South Australia, Australia; 11 Victorian Infectious Diseases Reference Laboratory, Doherty Institute, Melbourne, Victoria, Australia; 12 Pathology Queensland, Royal Brisbane and Women’s Hospital, Herston, Queensland, Australia; 13 Victorian Infectious Disease Service, Royal Melbourne Hospital, Melbourne, Victoria, Australia; NIH-National Institute for Research in Tuberculosis-ICER, INDIA

## Abstract

**Background:**

There are no national prevalence studies of *Strongyloides stercoralis* infection in Australia, although it is known to be endemic in northern Australia and is reported in high risk groups such as immigrants and returned travellers. We aimed to determine the seropositivity (number positive per 100,000 of population and percent positive of those tested) and geographical distribution of *S*. *stercoralis* by using data from pathology laboratories.

**Methodology:**

We contacted all seven Australian laboratories that undertake *Strongyloides* serological (ELISA antibody) testing to request de-identified data from 2012–2016 inclusive. Six responded. One provided positive data only. The number of people positive, number negative and number tested per 100,000 of population (Australian Bureau of Statistics data) were calculated including for each state/territory, each Australian Bureau of Statistics Statistical Area Level 3 (region), and each suburb/town/community/locality. The data was summarized and expressed as maps of Australia and Greater Capital Cities.

**Principal findings:**

We obtained data for 81,777 people who underwent serological testing for *Strongyloides* infection, 631 of whom were from a laboratory that provided positive data only. Overall, 32 (95% CI: 31, 33) people per 100,000 of population were seropositive, ranging between 23/100,000 (95% CI: 19, 29) (Tasmania) and 489/100,000 population (95%CI: 462, 517) (Northern Territory). Positive cases were detected across all states and territories, with the highest (260-996/100,000 and 17–40% of those tested) in regions across northern Australia, north-east New South Wales and north-west South Australia. Some regions in Greater Capital Cities also had a high seropositivity (112-188/100,000 and 17–20% of those tested). Relatively more males than females tested positive. Relatively more adults than children tested positive. Children were under-represented in the data.

**Conclusions/Significance:**

The study confirms that substantial numbers of *S*. *stercoralis* infections occur in Australia and provides data to inform public health planning.

## Author summary

*Strongyloides stercoralis*, a parasitic roundworm, is endemic in many countries world-wide. In Australia, groups at risk for strongyloidiasis include Aboriginal and/or Torres Strait Islander people, who acquired this parasite locally, and immigrants and returned travellers who acquired the infection outside Australia. We obtained deidentified results of ELISA IgG antibody tests for *Strongyloides* from diagnostic pathology laboratories during 2012 to 2016 and calculated the number of people who were positive at least once and the number who never had a positive result. We drew maps showing the number positive per 100,000 of population, the percent positive of those tested, and the number tested/100,000 for each region and the number positive in each suburb of residence according to the Australian Bureau of Statistics. The highest seropositivity (260-996/100,000 of population) was in Northern Australia, north-west South Australia and north-east New South Wales where many Aboriginal and Torres Strait Islander people live in remote communities. There were also some regions in Greater Capital Cities with a high number of people positive per 100,000 of population (112-188/100,000), likely reflecting higher populations of immigrants and returned travellers who were infected outside Australia.

## Introduction

*Strongyloides stercoralis* is a nematode parasite primarily of humans with a world-wide distribution, and is more common in areas of socioeconomic disadvantage [1]. The most recent global prevalence estimate (for 2017) was 8.1% corresponding to 613.9 million people infected [2].

In Australia, *S*. *stercoralis* is endemic in many Aboriginal and Torres Strait Islander communities [3–13]. Previous surveys show seropositivy rates of up to 58% of those tested (33/57) in the Kimberley region of Western Australia in 1986 [5] and 59% (220/372) in the East Arnhem region of the Northern Territory in 1989 [7]. The highest rate in a clinical setting was 51% (88/172) in East Arnhem Land in 2012–2016 [14]. Non-Aboriginal people with strongyloidiasis may have acquired the disease locally, such as workers or visitors in Aboriginal and Torres Strait Islander communities [15], or while overseas, such as returned international travelers [16,17], returned Armed Services personnel [18–20] and refugees and immigrants [20–22]. *S*. *stercoralis* is not a reportable infection and so the current prevalence in these high risk groups is uncertain.

*S*. *stercoralis* is a persistent infection due to internal autoinfection. Consequently, it is often present for many years before being diagnosed [18,21–23] and is usually a life-long infection unless treated effectively [18,22]. The chronic infection may be asymptomatic or exhibit mild intermittent symptoms primarily of the gut, respiratory system and skin [24,25]. In a seminal paper of chronic strongyloidiasis occurring in a group of Australian men deployed to South East Asia during World War 2, 27.5% (44/158) of whom were positive for *S*. *stercoralis*, Grove found that indigestion, urticaria, pruritus ani, diarrhoea and weight loss were significantly more frequently reported in infected men compared to uninfected men [18]. Pelletier who studied American men who had been subjected to similar conditions reported similar findings [26]. People with immune suppression, most often due to the administration of corticosteroid drugs, may develop disseminated disease and fatal illness if the infection is not diagnosed and treated [24,27,28]. Other immunosuppressant drugs (eg azathioprine, methotrexate, mycophenolate, cyclophosphamide, biological agents, chemotherapies) which also raise the risk of precipitating hyperinfection are increasingly being prescribed [27,29–31]. In addition, comorbidities (eg diabetes, alcoholism, hypochlorhydria, malnutrition, HTLV-1) are increasingly prevalent and also pose a significant risk for hyperinfection [24,32–35]. Patients may suffer serious secondary infections often caused by gut bacteria carried into the tissues by autoinfective larvae [24,28,36]. Current data on the burden of infection in Australia, especially in high risk populations, is needed to inform public health policy and planning [37].

In this study, our primary aim was to determine the number of persons seropositive for *Strongyloides* per 100,000 in the Australian population using routine laboratory data. Our secondary aims were 1) to describe *Strongyloides* seropositivity rates as the percent positive of those tested; 2) to examine the geographical distribution in Australian states and territories as well as geographical areas defined by boundaries set by the Australian Bureau of Statistics (ABS) (regions); 3) to investigate trends over time; and 4) to explore differences between sex and age groups nationally and for each state.

## Materials and methods

### Ethics statement

The project was approved by nine Ethics Committees: La Trobe University Human Ethics Committee EC00226 Project Number HEC 15–113, Central Australia Human Research Ethics Committee EC00155 Project Number HREC-16-382, Human Research Ethics Committee of the Northern Territory Department of Health and Menzies School of Health Research EC00153 Project Number 2016–2562, Aboriginal Health & Medical Research Council of New South Wales Ethics Committee EC00342 Project Number 1168/16, Western Sydney Local Health District Human Research Ethics Committee EC00152 Project Number 4904: LNR/16/WMEAD/452 LNR/SSA/16/WMEAD/460, Royal Brisbane & Women’s Hospital Human Research Ethics Committee EC00172 Project Number LNR/2018/QRBW/48092, Aboriginal Health Research Ethics Committee (South Australia) EC00185 Project Number 04-16-670, Central Adelaide Local Health Network Research Ethics Committee EC00192 Project Number HREC/16/RAH/172 CALHN R20160438 and Western Australia Aboriginal Health Ethics Committee EC00292 Project Number PR 698. Formal consent was obtained from all the Ethics Committees in writing.

### Study design and setting

We conducted a retrospective review of pathology laboratory data which had been recorded over the five years from 1 January 2012 to 31 December 2016 inclusive. It consisted of data from *Strongyloides* serology tests of people resident in all states and territories of Australia.

### Persons included in the study

#### Criteria for inclusion

The data include persons tested for *Strongyloides* by serology across all age groups and sexes with a known residential address in Australia (suburb/town, community/locality).

#### Criteria for exclusion

Persons with unknown or overseas residential addresses and those from Christmas Island, Norfolk Island, Keeling Island, and Lord Howe Island were excluded. Persons from Jervis Bay were included with New South Wales (NSW) data as it is surrounded by NSW, but considered as an “other territory” by the ABS.

### *Strongyloides* testing data

We requested *Strongyloides* testing data for the years 2012 to 2016 inclusive from all seven laboratories in Australia that undertake routine serological testing (that is, tests ordered by a health care professional). The data did not include any details about the reasons the tests were ordered, other helminth infections, other comorbidities or treatment. Some people had more than one test. Six of the laboratories provided *Strongyloides* serology data. One of those provided positive data only (Table 1). We were not able to obtain any information about the volume of testing from the private laboratory that did not contribute data to the study. It receives specimens from persons from every state and territory in Australia.

**Table 1:** Contribution of each laboratory to the *Strongyloides* serology data, 2012–2016, number of people tested and percentage of the data contributed by each laboratory to each state or territory of residence.

State /territory of residence	ACT	NSW	NT	QLD	TAS	VIC	WA	SA	Total
Laboratory location	n (%)	n (%)	n (%)	n (%)	n (%)	n (%)	n (%)	n (%)	n (%)
^1^NSW	296 (13.8)	9,230 (44.5)	7 (0.1)	2,504 (18.6)	1 (0.0)	65 (0.3)	9 (0.1)	173 (14.1)	12,285 (15.0)
^1^QLD	108 (5.0)	50 (0.2)	18 (0.3)	4,293 (31.9)	0 (0.0)	35 (0.1)	13 (0.1)	8 (0.7)	4,525 (5.5)
^2^QLD P1	1,728 (80.6)	11,307 (54.6)	58 (0.8)	6,566 (48.8)	294 (10.0)	7,075 (30.2)	717 (6.7)	486 (39.6)	28,231 (34.5)
^1^VIC	9 (0.4)	119 (0.6)	355 (5.0)	85 (0.6)	2,641 (89.9)	16,276 (69.4)	22 (0.2)	29 (2.4)	19,533 (23.9)
^1^WA	4 (0.2)	11 (0.1)	6,593 (92.5)	10 (0.1)	3 (0.1)	8 (0.0)	9,931 (92.8)	12 (1.0)	16,572 (20.3)
*Subtotal	2,145 (100)	20,717(100)	7,031 (98.6)	13,458(100)	2,939 (100)	23,456 (100)	10,692(99.9)	708 (57.7)	81,146 (99.2)
§^1^SA	0 (0.0)	2 (0.0)	97 (1.4)	0 (0.0)	0 (0.0)	5 (0.0)	8 (0.1)	519 (42.3)	631 (0.8)
**Total	2,145 (100)	20,719 (100)	7,128 (100)	13,458 (100)	2,939 (100)	23,461 (100)	10,700 (100)	1,227 (100)	81,777 (100)
% of data	2.6	25.3	8.7	16.5	3.6	28.7	13.1	1.5	100

^1^Government laboratory.

^2^Private laboratory situated in QLD.

*Used for calculation of percent positive of those tested.

**Used for calculation of the number positive per 100,000 of population.

§The laboratory in SA contributed positive results only. ACT = Australian Capital Territory; NSW = New South Wales; NT = Northern Territory; QLD = Queensland; TAS = Tasmania; VIC = Victoria; WA = Western Australia; SA = South Australia.

### Variables

The *Strongyloides* serology data included the following fields: de-identified unique identifier, sex (male, female, unknown), age (years), suburb/town/community/locality of residence, postcode, state, date and result of test. For reporting, age was categorized as 0–4, 5–14, 15–24, 25–34, 35–44, 45–54, 55–64, 65–74, ≥75 years or unknown if age was missing.

### Measurements

#### *Strongyloides* serology methods

A list of the ELISA method and cutoff values for each laboratory are given in S1 Table. Three enzyme-linked immuno-sorbent assays (ELISA) that detect IgG antibodies to *Strongyloides* were used: Bordier ELISA (Bordier Affinity Products SA, Crissier, Switzerland) based on somatic antigens of *S*. *ratti* (three laboratories), an in-house method, also based on *S*. *ratti* somatic antigens (one laboratory), IVD ELISA (DRG Instruments GmbH, Marburg, Germany) based on somatic antigens of *S*. *stercoralis* infective larvae (two laboratories) one of which transitioned from an in-house method based on *S*. *ratti* antigens during 2012 [38,39]. The equivocal range represents an overlap in results between standard negative and standard positive sera. All Australian laboratories performing *Strongyloides* serology participated in an informal quality assurance programme. For the purposes of the statistical analysis, only positive results were considered as positive and equivocal results were included with the negative results as negative to avoid overestimation of the positive results. All these methods detect *Strongyloides* at the generic level and are not specific for *S*. *stercoralis*. However, *S*. *stercoralis* is the species that is prevalent in Australia. In a global genotyping survey of *S*. *stercoralis* and *S*. *fuelleborni*, two specimens of *S*. *fuelleborni* were said to come from Australia [40]. These samples actually came from Senegal and Guinea-Bisseau in Africa [41].

#### Terminology

In this study, seropositivity refers to both the number of people positive for *Strongyloides* by serological testing per 100,000 of population and the percent positive of those tested.

### Bias

The persons included were those for whom a *Strongyloides* serology test was requested by a health care professional and therefore do not represent a random sample of the total population of Australia and so are not generalizable to the total population.

### Study size

The *Strongyloides* serology test data in Australia for the five years 2012 to 2016 was not complete, as one private laboratory did not contribute data to the project. With the exception of the government laboratory in SA that provided positive serology data only, the laboratories provided de-identified positive, negative, and equivocal serology data. More than one laboratory contributed data for each state and territory of residence. Table 1 shows the contribution of each laboratory to the data for each state or territory of residence.

### Data access and cleaning methods

**Fig 1:**
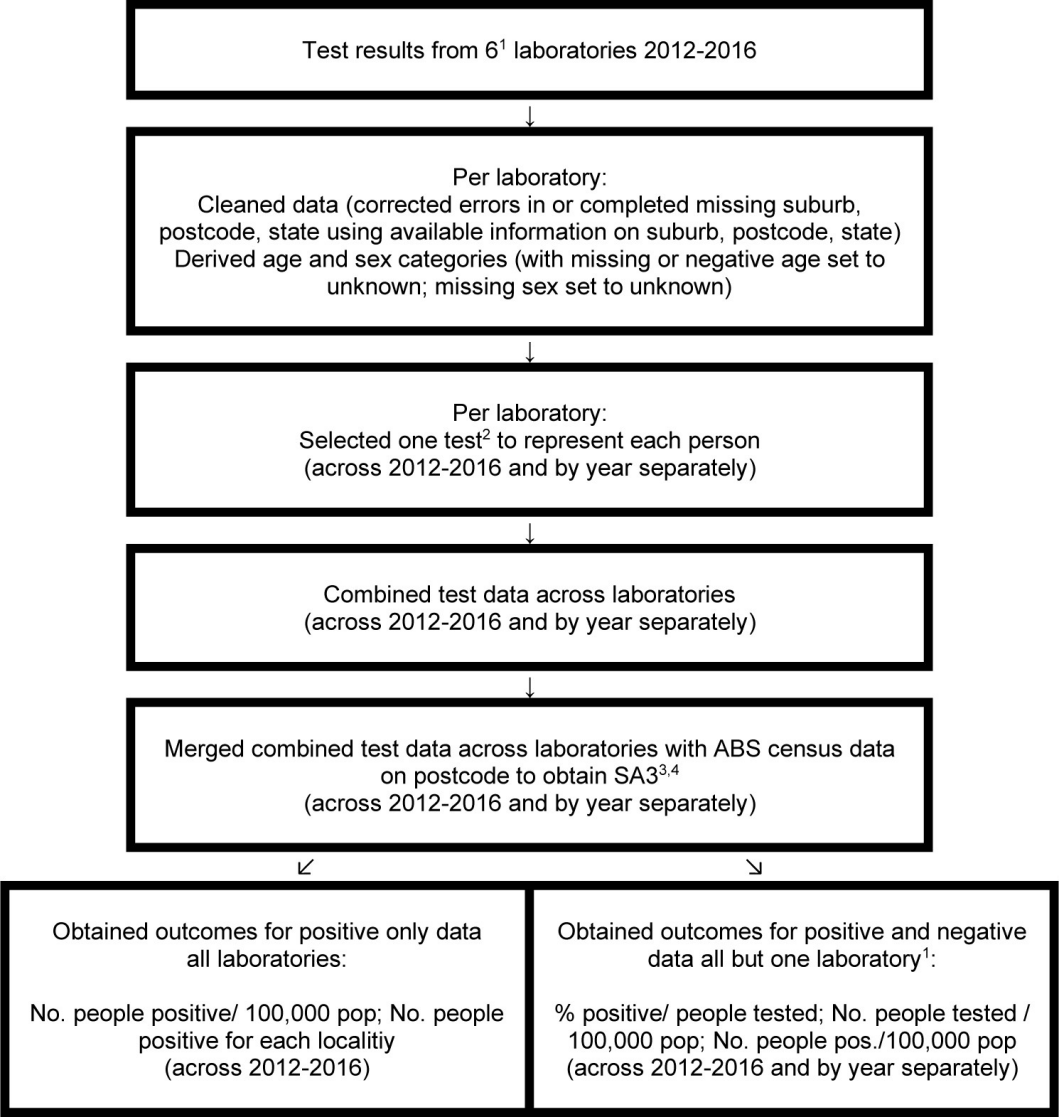
Flowchart: data processing steps of *Strongyloides* IgG ELISA serology test results. ^1^Positive and negative results for 5 laboratories, positive only results for laboratory in South Australia. ^2^For those who tested positive at least once, the selected test was the first positive test. For those who tested only equivocal or negative, the selected test was the first equivocal or negative test. ^3^SA3 was based on 2011 ABS census data. When merging ABS data with the combined test data across 2012–2016, ABS data with population sizes for 2012–2016 for each age and sex combination was used. When merging ABS data with the combined test data by year, ABS data with population sizes for each year 2012–2016 across all ages and sex combinations was used. ^4^The resulting merged combined data set excluded overseas, remote island (Christmas Island, Norfolk Island, Lord Howe Island, Keeling Island), and missing suburbs. For those postcodes that were linked to more than one SA3, the SA3 was selected manually using the person’s postcode, suburb and state information together with the ABS postcodes map and Google maps. ABS = Australian Bureau of Statistics; SA3 = Statistical Area Level 3 (region defined by ABS, 2011); pos = positive; pop = population.

A summary of the data processing and outcomes is given in Fig 1. The serology data set from each participating pathology laboratory was cleaned by JS and SB by identifying then correcting errors in postcode, suburb, and/or state/territory. This included assigning the most likely suburb, postcode or state in records where where this information was missing where there was sufficient information. Where postcodes crossed state borders, the state given in some records needed correction. As the names of many Aboriginal communities in the NT, SA and WA have recently changed, where records included the old name this was corrected to the current one.

### Statistical methods

Data were processed using Statistical Software Stata/SE 15.1 (College Station, TX: StataCorp LLC).

Persons with one or more positive results were classified as positive and data from their first positive test result only was included in the data set. Persons with only negative or equivocal results were classified as negative and the data from their first test result only was included. The data from the laboratories was combined and merged with Australian Bureau of Statistics (ABS) 2011 census data on postcode [42] to obtain the Statistical Area Level 3 (SA3) for each record. Each SA3 represents a region or one or two adjacent regions within each state. For those postcodes that were linked to more than one SA3, JS manually selected the most appropriate single SA3 using the person’s postcode, suburb, and state information together with the ABS postcodes map [43] and Google maps [44] to locate the suburb. SA3 Special Purpose Codes were not included.

From the data, we reported the number of persons with at least one positive test result and the number of persons with at least one test result. We derived the number of persons with at least one positive test result per 100,000 of population using the (average of) population sizes projected for 2012–2016 from the 2011 census of the ABS for each state and each statistical area level 3 (SA3) [42] from the total data. Similarly, after excluding the data from the laboratory that provided positive data only, we derived the percent of people positive per those tested, the number tested per 100,000 of population and, for comparison, the number positive per 100,000 for Australia, each state/territory, for each SA3, for each year of testing and for each sex and age group. Clopper-Pearson exact two-sided 95% confidence intervals (CI) were calculated on proportions. In addition, maps of seropositivity (the number positive per 100,000 of population and the percent positive of those tested) for each ABS SA3 were created using the ABS shapefile for 2011 [42] and the Tableau mapping package. Geograpical coordinates of the suburbs, towns, communities or localities of residence of people positive for *Strongyloides* were obtained from Google Maps and plotted on a map using Tableau mapping software. Greater Capital Cities were not included in this map, as the SA3 maps gave sufficient detail for closely populated areas.

The ABS shapefile is provided under Creative Commons Attribution 4.0 International. Tableau uses Mapbox and OpenStreetMap maps. This is acknowledged by the text: © Mapbox and © OpenStreetMap on each map. Mapbox.js is an open source project.

## Results

### Participants and descriptive data

The data represents 81,777 people who were tested for *Strongyloides* by serology in Australia during the five years 2012–2016 (Table 1). After excluding the 631 people from the SA laboratory, there were 81,146 people who underwent *Strongyloides* serology testing for whom both positive and negative results were available. Of these 46.2% were female, 53.5% male, 0.3% whose sex was unknown (S6 Table); 3.4% were 0–4 years, 11.3% 5–14 years, 15.4% 15–24 years, 18.5% 25–34 years, 15.9% 35–44 years, 13.1% 45–54 years, 10.7% 55–64 years, 7.0% 65–74 years, 4.5% ≥75 years and 0.1% whose age was unknown (S7 Table).

### Positive data from all six laboratories

#### Australia

The total number of people who were positive in all states and territories was 7,497. The projected average population size for 2012–2016 was 23,465,538 (ABS data [42]) so 32 (95% CI: 31, 33) people per 100,000 of population were positive.

#### States and territories

The number of people positive per 100,000 of population for each state and territory is given in S2 Table. The SA laboratory that contributed positive data only to the study did not include tests for any residents of the ACT, TAS or QLD and included very few tests for NSW, VIC and WA, so the number of people positive per 100,000 for these states and territories was the same as for the data excluding the SA laboratory (Table 2). The number of people positive per 100,000 for Australia, NT and SA was substantially higher when including the SA laboratory data. The figures are given in the footnote to Table 2. The number of positives per 100,000 for the NT was an order of magnitude higher than that of any of the other states or territories.

**Table 2:** *Strongyloides* serology: summary of seropositivity for each state and territory, 2012–2016, excluding data from the laboratory that provided positive data only*.

State /territory of residence	No. of people tested	No. of people positive	% Positive of those tested (95% CI)	Average annualized population	No. tested /100,000 (95% CI)	No. positive /100,000 (95% CI)
ACT	2,145	117	5.4 (4.5, 6.5)	389,502	551 (528, 574)	30 (25, 36)
NSW	20,717	1,813	8.8 (8.4, 9.1)	7,513,103	276 (272, 280)	24 (23, 25)
*NT	7,031	1,087	15.5 (14.6, 16.3)	242,180	2,903 (2,837, 2,971)	449 (423, 476)
QLD	13,458	1,431	10.6 (10.1, 11.2)	4,712,802	286 (281, 290)	30 (29, 32)
TAS	2,939	117	4.0 (3.3, 4.8)	514,041	572 (551, 593)	23 (19, 27)
VIC	23,456	1,521	6.5 (6.2, 6.8)	5,902,834	397 (392, 402)	26 (24, 27)
WA	10,692	723	6.8 (6.3, 7.3)	2,505,342	427 (419, 435)	29 (27, 31)
*SA	708	57	8.1 (6.2, 10.3)	1,685,734	42 (39, 45)	3 (3, 5)
Australia	81,146	6,866	8.5 (8.3, 8.7)	23,465,538	346 (343, 348)	29 (29, 30)

*When the positive data from the SA laboratory that provided positive data only was included, the number positive per 100,000 of population for Australia was 32 (95% CI: 31, 33), for the NT was 489 (95% CI: 462, 517) and for SA was 34 (95% CI: 31, 37). CI = confidence interval, ACT = Australian Capital Territory; NSW = New South Wales; NT = Northern Territory; QLD = Queensland; TAS = Tasmania; VIC = Victoria; WA = Western Australia; SA = South Australia.

#### Regions (ABS SA3s)

**Fig 2:**
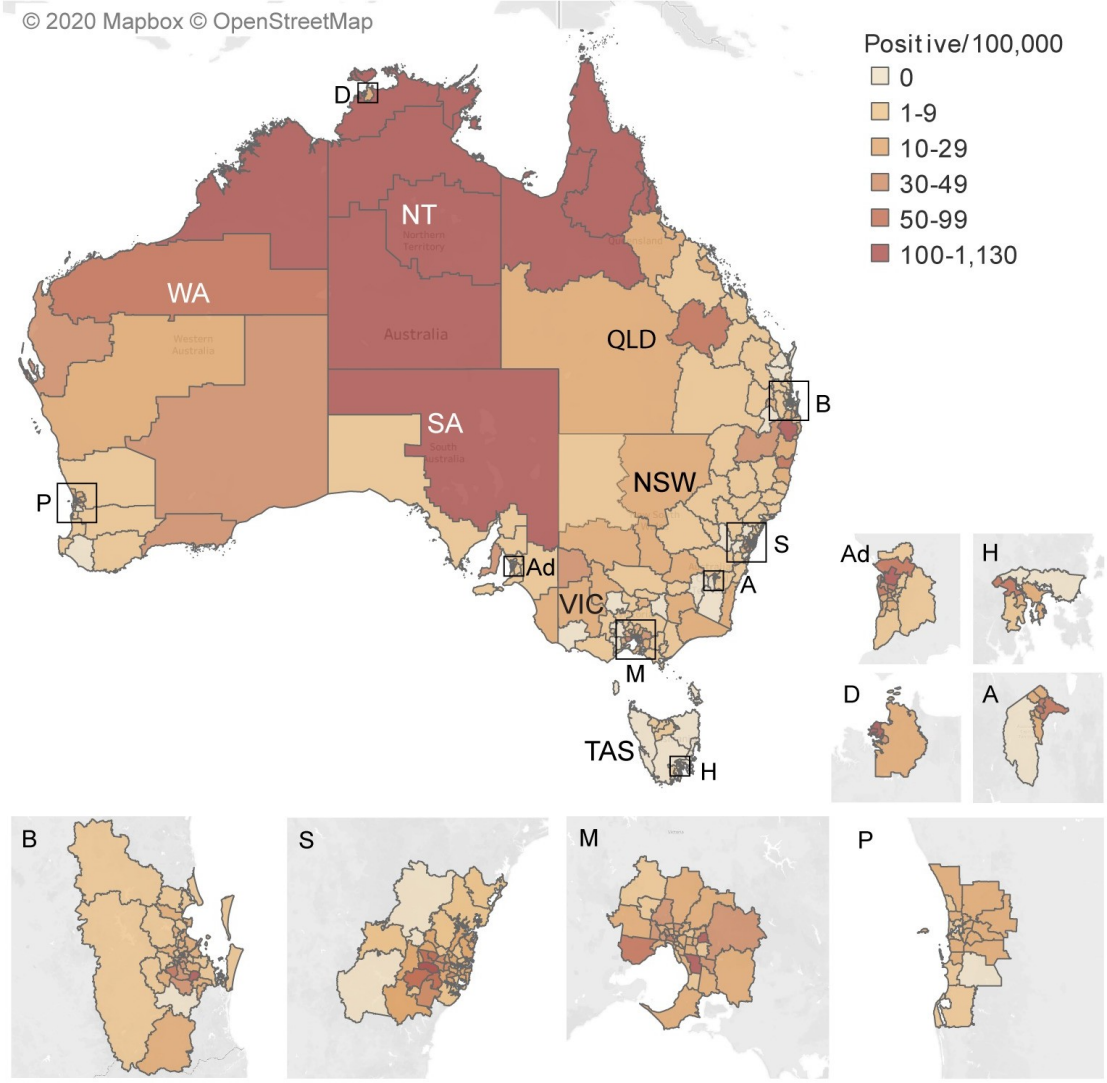
*Strongyloides* serology: map^1^ showing the number of people positive per 100,000 population, for each ABS statistical area level 3 (region), 2012–2016, Australia and greater capital cities, including the positive data from all six laboratories. ^**1**^This map was created using, our data, Tableau software, an ABS shapefile and a Mapbox base map. A = ACT; Ad = Adelaide; B = Brisbane; D = Darwin; H = Hobart; M = Melbourne P = Perth; S = Sydney; NSW = New South Wales; NT = Northern Territory; QLD = Queensland; SA = South Australia; TAS = Tasmania; VIC = Victoria; WA = Western Australia.

The number of people positive per 100,000 of the population for each region is given in S3 Table and mapped in Fig 2. In general, the SA3s with the greatest number positive per 100,000 of population were in northern QLD, the whole of the NT except for outer Greater Darwin, the north of WA, the north-west of SA and the north-east of NSW. There were also SA3s with high seropositivity in the Greater Capital Cities except Perth.

#### Suburbs, towns, communities and localities

The number of people positive in each suburb, town, community or locality excluding the ACT and Greater Capital Cities is shown in S1 Fig. This shows that *Strongyloides*-positive residents are geographically widespread in Australia. The suburbs/towns/communities/localities with the greatest number of people positive for *Strongyloides* were in northern WA, NT and QLD, north-east NSW and north-west SA. Although the Outback-North and East SA3 in SA covers are very large area of land, S1 Fig shows that the main area of seropositivity was in the north-west, close to the border with the NT.

### Positive and negative data, excluding data from the laboratory that provided positive data only

#### Australia

Positive and negative data was available from five laboratories for 81,446 people of whom 6,866 were positive. The percent positive of those tested and the number tested per 100,000 of population are given in Table 2. The number positive per 100,000 of population is given for comparison with the calculation based on positive data from all laboratories.

#### States and territories

The percent positive of those tested for each state and territory is presented in Table 2. We have also provided the number positive per 100,000 of population using this data set for comparison with the positive only data from all six laboratories (S2 Table). The main differences between Table 2 and S2 Table are also provided as a footnote.

#### Regions

**Fig 3:**
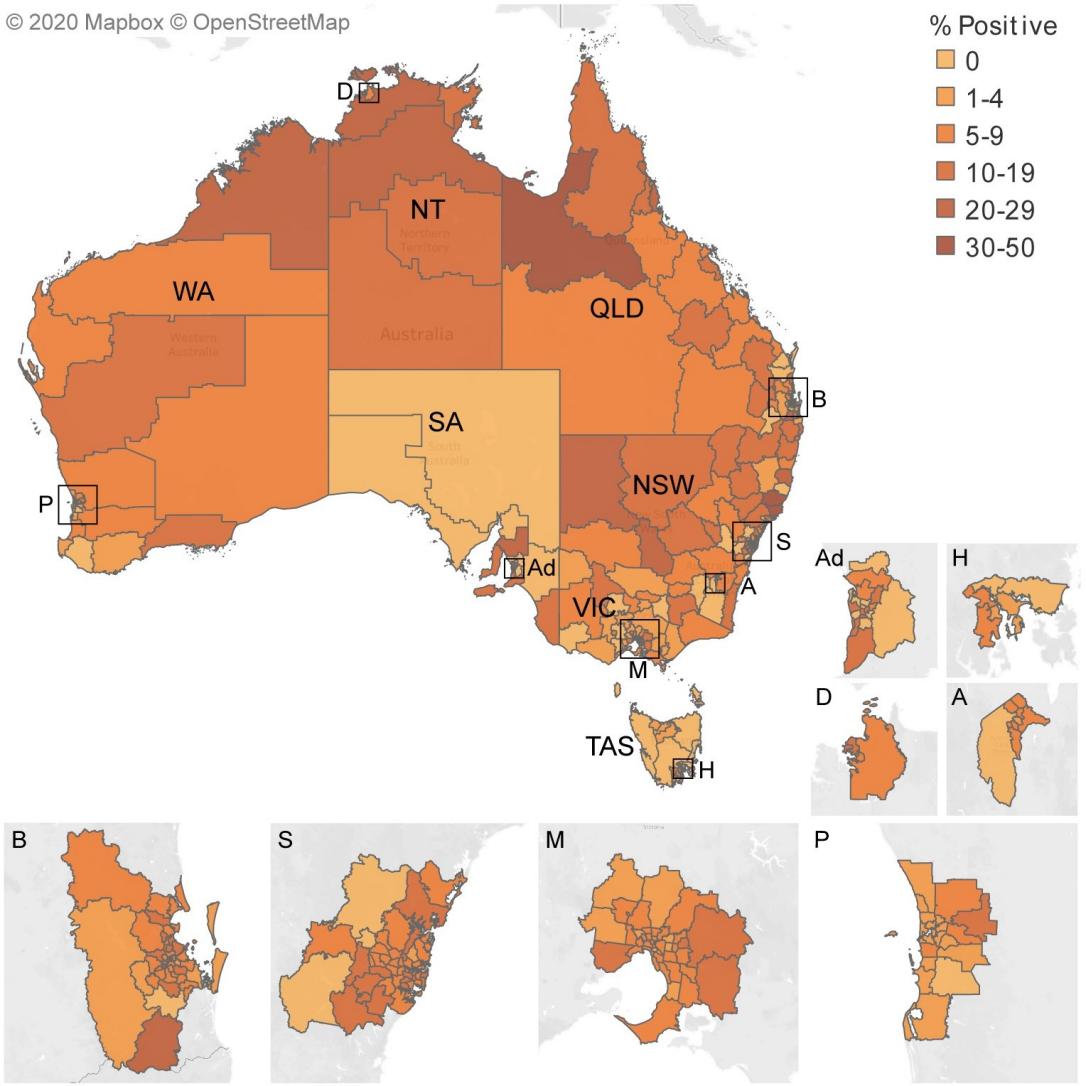
*Strongyloides* serology: map^1^ showing percent positive of those tested, 2012–2016, (excluding data from the laboratory that provided positive data only^2^) for each ABS statistical area level 3 (region) in each state and Greater Capital City. ^**1**^This map was created using our data, Tableau software, an ABS shapefile and a Mapbox base map. ^2^Most of SA shows 0% positive because there was no negative data from the laboratory in SA. The % positive for the two southernmost SA3s in NT are likewise underestimated. A = ACT; Ad = Adelaide; B = Brisbane; D = Darwin; H = Hobart; M = Melbourne; P = Perth; S = Sydney; NSW = New South Wales; NT = Northern Territory; QLD = Queensland; SA = South Australia; TAS = Tasmania; VIC = Victoria; WA = Western Australia.

The percent positive of those tested in each SA3 is given in S4 Table and mapped in Fig 3. The number tested per 100,000 is given in S4 Table and mapped in S2 Fig. In 20 SA3s, the number of people positive per 100,000 of population was >100/100,000. Seven of these were in Greater Capital Cities. Apart from Richmond Valley Hinterland in north-east NSW and Outback-North and East in SA, the SA3s with the highest seropositivity were in the north of Australia. In six SA3s both the number of people positive per 100,000 of population was >100/100,000 and percent positive of those tested was >20%. They were Kimberley in WA (996/100.000 (95% CI 898, 1102) and 22.3% (95% CI 20.4, 24.4) respectively), Outback-North (589/100,000 (95% CI 509, 677) and 40.4% (95% CI 36.0, 45.0) respectively), Port Douglas-Daintree (146/100,000 (95% CI 85, 234) and 22.1% (95% CI 13.4, 33.0) respectively) and Innisfail-Cassowary Coast (740/100,000 (95% CI 654, 835) and 21.1% (95% CI 18.9, 23.5) respectively) in QLD, and Daly-Tiwi-West Arnhem (747/100,000 (95% CI 602, 853) and 27.4% (95% CI 23.5, 31.6) respectively) and Katherine (284 (95% CI 212, 360) and 25.5% (95% CI 20.0, 31.7) respectively) in the NT. A further twenty-one SA3s with high seropositivity (more than 50 positive per 100,000 of population) (95%CI: 31, 33) were located in Central Highlands in QLD, Coffs Harbour in north-east NSW, Pilbara in WA as well as in Greater Capital Cities except Perth, WA.

S2 Fig shows that in general testing was highest in those SA3s that had the greatest seropositivity, in northern Australia.

#### Results by year

**Fig 4:**
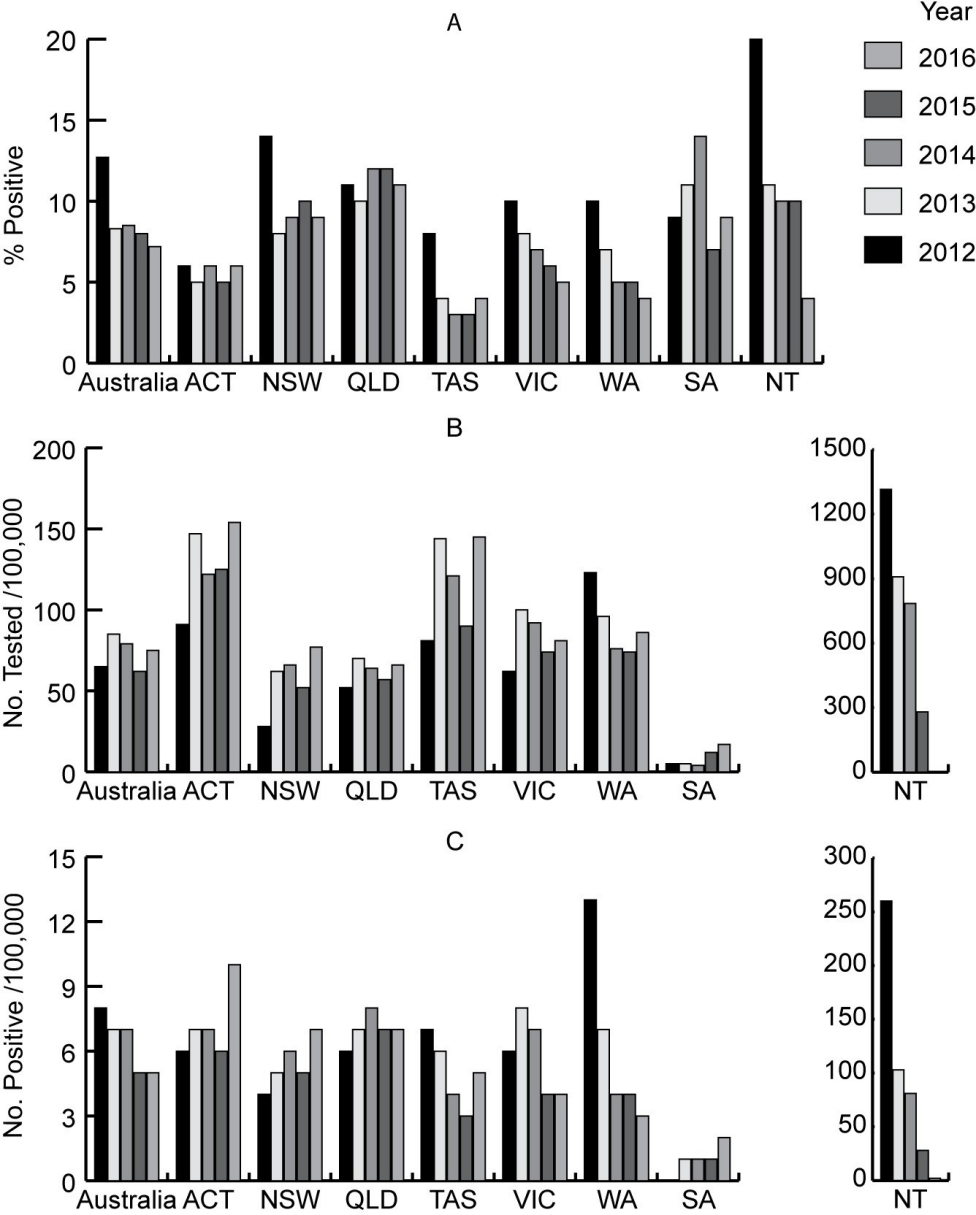
*Strongyloides* serology: number of people tested and positive in each year, 2012−2016^1^, (excluding the data from the SA laboratory that provided positive results only^2^): Australia and states and territories. **A. Percent positive of those tested. B. Number of people tested per 100,000 of population. C. Number of people positive per 100,000 of population.** ^1^ Number of people positive per 100,000 of population. ^**2**^The values for SA in B and C are considerably underestimated because of the exclusion of data from the laboratory in SA. ACT = Australian Capital Territory; NSW = New South Wales; NT = Northern Territory; QLD = Queensland; SA = South Australia; TAS = Tasmania; VIC = Victoria; WA = Western Australia.

The percent positive of those tested, the number of people tested per 100,000 of population and the number positive per 100,000 for Australia and each state and territory in each year 2012–2016 (excluding data from the laboratory that provided positive data only) are given in Fig 4 and S5 Table. There was an overall decline in the percent positive of those tested for Australia over the 5 year period (from an average of 12.7% (95% CI 12.1,13.2) in 2012 to 7.2% (95% CI 6.8, 7.5)) in 2016. There was also a sharp decline in the number tested/100,000 of population in the NT over the five year period.

#### Sex and age group

The overall results for Australia for each sex and age group are given in Table 3. The frequency data for each state and territory for sex are given in S6 Table and S3 Fig and for age group, in S7 Table and S4 Fig. The low number of tests for SA is due to the exclusion of data from the SA laboratory that provided positive results only. For Australia as a whole, there were relatively more males than females but this trend was not consistent across states and territories The percent positive of those tested and the number of people positive per 100,000 of population increased with age for Australia and all states and territories. Positivity and the number tested per 100,000 of population increased with age up to the 25–34 years age group (S7 Table, S4 Fig).

**Table 3:** *Strongyloides* serology results for each sex and age group for Australia 2012–2016 (excluding data from the SA laboratory that provided positive data only).

	No. of people tested	No. of people positive	% Positive of those tested (95% CI)	Average annualized population	No. of people tested /100000 (95% CI)	No. of people positive /100000 (95% CI)
**Sex**						
Female	37,476	3,053	8.1 (7.9, 8.4)	11,803,929	317 (314, 321)	26 (25, 27)
Male	43,451	3,787	8.7 (8.5, 9.0)	11,661,609	373 (369, 376)	33 (31, 34)
Unknown	219	26	11.9 (7.9, 16.9)			
**Age (years)**						
0–4	2,788	84	3.0 (2.4, 3.7)	1,537,150	181 (175, 188)	5 (4, 7)
5–14	9,209	329	3.6 (3.2, 4.0)	2,901,067	317 (311, 324)	11 (10, 12)
15–24	12,532	967	7.7 (7.3, 8.2)	3,129,417	400 (393, 408)	31 (29, 33)
25–34	14,979	1,241	8.3 (7.8, 8.7)	3,455,515	433 (427, 440)	36 (34, 38)
35–44	12,893	1,206	9.4 (8.9, 9.9)	3,216,414	401 (394, 408)	37 (35, 40)
45–54	10,619	1,160	10.9 (10.3, 11.5)	3,102,036	342 (336, 349)	37 (35, 40)
55–64	8,721	931	10.7 (10.0, 11.3)	2,681,694	325 (318, 332)	35 (33, 37)
65–74	5,715	563	9.9 (9.1, 10.7)	1,931,354	296 (288, 304)	29 (27, 32)
≥75	3,614	368	10.2 (9.2, 11.2)	1,510,891	239 (231, 247)	24 (22, 27)
Unknown	76	17	22.4 (13.6, 33.4)			
Total	81,146	6,866	8.5 (8.3, 8.7)	23,465,538	346 (343, 348)	29 (29, 30)

CI = confidence interval.

## Discussion

This study represents the most comprehensive analysis of *Strongyloides stercoralis* serology testing data ever undertaken in Australia. Our finding of 32 positive cases per 100,000 population for Australia, equivalent to 0.032% (0.032/100 of the population) is approximately three times the estimate of 0.01% by Buonfrate et al [2] for the Australian population and is similar to that of Italy, Belgium, France, Malta, Japan and New Zealand [2]. The true figure for Australia would be higher than this because our calculation represents only those tested. *S*. *stercoralis* infections are widespread as cases were detected across all states and territories and in most regions.

The high seropositivity in regions across northern Australia, north-east NSW and north-west SA confirming earlier work [5–7,9–14,45–47], likely reflected infections mainly in Aboriginal and Torres Strait Islander Australians who were infected in Australia. The high seropositivity in regions in Greater Capital Cities likely reflected mainly people who were infected in other countries: immigrants and returned international travellers including Armed Services personel [17–19,21,22,48]. However, it was not known to what extent Aboriginal and Torres Strait Islander people resident in Greater Capital Cities had acquired *S*. *stercoralis* infection while previously living in or visiting communities where it is endemic [13]. Due to effective sanitation, transmission is less likely in the Australian urban environment [49].

Other similar countries with an endemic population and an immigrant population infected with *S*. *stercoralis* include the USA, Spain and Italy [25,50–52], whereas in Canada and north European countries, *S*. *stercoralis* infections are limited to immigrants and returned international travellers [16,53,54]. In the USA, a meta-analysis of community surveys in endemic regions found three percent positive, lower than in endemic regions of Australia [55], and in Spain and Italy, nearly all infected people were in older age groups [25,52], suggesting that transmission was rare.

Although the greatest number of people positive for *Strongyloides* was in NSW, the state with the largest population, the NT, the state with with the smallest population had by far the greatest number positive per 100,000 of the population and the greatest percentage positive of people tested. This higher infection rate was presumably reflecting a majority of cases within the Aboriginal and/or Torres Strait Islander population that reside in small remote communities where *S*. *stercoralis* is known to be endemic [8,14,20,45].

The high rate of seropositivity in Central Australia (Alice Springs and Barkly region in the southern part of the NT, and adjacent communities in the Outback—North and East region in SA and in Goldfields region in WA) coincides with a high prevalence of HTLV-1 infection [47,56]. Complicated strongyloidiasis has been reported in coinfections with HTLV-1 leading to death in some patients [47], and conversion of asymptomatic HTLV-1 to clinical HTLV-1 [57] has also been reported in patients with *S*. *stercoralis* infection. Further research is needed to elucidate the relative risks of coinfection with these pathogens in this area.

Children (0–4 years and 5–14 years) were under-represented in the serology data even though there is considerable seropositivity in children. This is likely because venipuncture is more difficult in children. *S*. *stercoralis* has been associated with malnutrition and hypokalaemia in very young children [58] and carriage into adulthood [13]. The “community children’s de-worming program” for northern Australia recommends treating children with single dose oral albendazole twice a year (in line with WHO guidelines) and has been effective against hookworm [59–61]. Although albendazole is used as second line treatment for strongyloidiasis, repeated doses are necessary [62]. A routine *Strongyloides* serology test that utilises finger-prick blood would make routine testing and treatment of children possible and provide improved epidemiological data. The increasing seropositivity with age followed trends in other parts of the world [63–66].

### Limitations

This paper is based on aggregated de-identified laboratory data and as such the reason for testing was not known, nor the outcome for the patients. Therefore the data are not representative of prevalence rates in the total population. The data did not distinguish between the various at-risk categories of people, so we made assumptions based on the geographic origin of the test and the known location of the two main at risk populations, Aboriginal and Torres Strait Islander Australians and immigrants.

The number of people reported positive in SA3s where there is a hospital may be overstated as in-patients may have used the address of the hospital instead of their usual residential address. This was particularly evident for the Darwin suburb of Tiwi, the location of the Royal Darwin Hospital, and affected the Darwin Suburbs SA3 figures. This would not have affected the overall results for the NT.

The serology data is incomplete. One major laboratory did not contribute data to the study. The number of people in the missing data is unknown. It included tests from all states and territories, predominantly QLD and northern NSW [67], so the results from these areas are likely to be underestimated. The data from the NT in this study showed a sharp decline in numbers of people tested between 2012 and 2016 (Fig 4, S5 Table). It is likely that the missing data is at this laboratory. The SA laboratory which provided positive data only, contributed the only positive data for the Outback—North and East region of SA and most of the positives for the Barkly region and more than half of the positives for the Alice Springs region of NT. These regions are recognized locally as areas of high endemicity for *S*. *stercoralis*. Because it was necessary to exclude this data for estimates of the percent positive of those tested for these regions, as well as for SA and the NT, these measures are likely underestimated, but this did not affect the number positive per 100,000 of population.

Some people may have been tested by more than one laboratory, in which case they would appear in the data twice with two different unique identifiers and a few people may have changed their name or date of birth between tests. However, this is likely to be a small number and therefore a minor influence on the overall result.

ELISA based IgG Strongyloides serology is currently the most widely used diagnostic test for *Strongyloides* in Australia because of the convenience of collecting and transporting serum to laboratories and the relatively high test sensitivity except in early infection and when the patient is immunosuppressed. False positives can occur with some other helminth infections acquired outside Australia [39]. The sensitivity and specificity of the tests used in this study when compared to a gold standard of larval microscopy have been estimated as follows: IVD ELISA: sensitivity 91%, specificity 99%; Bordier ELISA: sensitivity 90%, specificity 98%; in-house ELISA: sensitivity 93%, specificity 95% [38,39].

## Conclusions

Overall, *Strongyloides* seropositivity in Australia for the 5 years 2012–2016 was low at 32 per 100,000 of population, and 8.5% of those tested in all states and territories.

*S*. *stercoralis* was detected in all states and territories. The number of people positive per 100,000 of population and the percent positive of those tested was highest in regions in the NT, the north of WA, north QLD, north-east NSW, and north-west SA (no percent positive estimate), where a high proportion of the population live in Aboriginal and/or Torres Strait Islander communities. It was also high in some regions in Greater Capital Cities where there is a large immigrant population.

National guidelines for controling strongyloidiasis in Aboriginal and Torres Strait Islander communities with a focus on raising awareness in communities, improving health facilities and supporting and educating health staff would greatly assist in controling this and other infectious diseases [68–70]. Population-based serosurveys would assist in determining the true prevalence in high risk populations and provide data to inform public health planning.

## Supporting information

S1 Fig *Strongyloides* serology: map^1^ of number of people positive for each suburb, town, community or locality^2^, 2012–2016, including data from all six laboratories. ^**1**^This map was created using our data, Tableau software and a Mapbox base map. ^2^The ACT and greater capital cities have been omitted. The ranges of numbers positive are shown by colour and size of the dots. ACT = Australian Capital Territory; NSW = New South Wales; NT = Northern Territory; QLD = Queensland; SA = South Australia; TAS = Tasmania; VIC = Victoria; WA = Western Australia.
(TIF)

S2 Fig *Strongyloides* serology: map^1^ of number of people tested for *Strongyloides* per 100,000 of population, for each Australian Bureau of Statistics Statistical Area level 3, 2012–2016, excluding data from the laboratory that provided positive data only^2^. ^**1**^This map was created using our data, Tableau software, an ABS shapefile and a Mapbox base map.^2^This accounts for the low number of tests in South Australia. ACT = Australian Capital Territory; NSW = New South Wales; NT = Northern Territory; QLD = Queensland; SA = South Australia; TAS = Tasmania; VIC = Victoria; WA = Western Australia.
(TIF)

S3 Fig *Strongyloides* serology: frequency data for females and males for Australia and all states and territories 2012–2016 (excluding the data from the SA laboratory that provided positive results only^1^). **A. Percent positive of those tested. B. Number tested per 100,000 of population. C. Number positive per 100,000 of population.** ^1^The values for SA in B and C are considerably underestimated because of the exclusion of data from the laboratory in SA. ACT = Australian Capital Territory; NSW = New South Wales; NT = Northern Territory; QLD = Queensland; SA = South Australia; TAS = Tasmania; VIC = Victoria; WA = Western Australia.
(TIF)

S4 Fig *Strongyloides* serology: frequency data by age group (years) for Australia and each state or territory of residence, 2012–2016 (excluding the data from the SA laboratory that provided positive results only^1^). **A. Percent positive in each age group. B. Number of people tested per 100,000 of population in each age group and each state. C. Number of people positive per 100,000 of population for each age group.** ^1^The values for SA in B and C are considerably underestimated because of the exclusion of data from the laboratory in SA. ACT = Australian Capital Territory; NSW = New South Wales; NT = Northern Territory; QLD = Queensland; SA = South Australia; TAS = Tasmania; VIC = Victoria; WA = Western Australia.
(TIF)

S1 Table Strongyloides serology: cutoff values for the ELISA IgG serum tests, as provided by each laboratory. ^1^Ratio of the optical density of the test/optical density of the weak positive control. ^2^OD = optical density of the test solution. ^3^The in-house *S*. *ratti* assay at the WA laboratory was replaced by IVD ELISA during 2012. NSW = New South Wales; QLD = Queensland; SA = South Australia; VIC = Victoria; WA = Western Australia; P1 Private laboratory 1. (P2 did not contribute data to the study).
(DOCX)

S2 Table *Strongyloides* serology: number of people positive for *Strongyloides stercoralis* infection per 100,000 of the population for Australia and each state and territory, 2012–2016, data from all six laboratories. CI = confidence interval, ACT = Australian Capital Territory; NSW = New South Wales; NT = Northern Territory; QLD = Queensland; TAS = Tasmania; VIC = Victoria; WA = Western Australia; SA = South Australia.
(DOCX)

S3 Table *Strongyloides* serology: number of people positive per 100,000 of population for each Australian Bureau of Statistics Statistical Area Level 3 (SA3), 2012–2016, data from all six laboratories. ACT = Australian Capital Territory; NSW = New South Wales; NT = Northern Territory; QLD = Queensland; SA = South Australia; TAS = Tasmania; VIC = Victoria; WA = Western Australia.
(DOCX)

S4 Table *Strongyloides* serology: percent of people positive of those tested and number tested per 100,000 of population for each Australian Bureau of Statistics Statistical Area Level 3 (SA3), 2012–2016, excluding data from the laboratory that provided positive data only^1^. ^1^This accounts for the low number of tests in South Australia. ACT = Australian Capital Territory; NSW = New South Wales; NT = Northern Territory; QLD = Queensland; SA = South Australia; TAS = Tasmania; VIC = Victoria; WA = Western Australia.
(DOCX)

S5 Table *Strongyloides* serology: data by year, 2012−2016^1^, for Australia and each state and territory, excluding data from the laboratory that provided positive data only. **The number of people were calculated separately for each year.** ^1^The number of people were calculated separately for each year so a person who was tested in more than one year appears more than once in the data. ACT = Australian Capital Territory; NSW = New South Wales; NT = Northern Territory; QLD = Queensland; SA = South Australia; TAS = Tasmania; VIC = Victoria;, WA = Western Australia.
(DOCX)

S6 Table *Strongyloides* serology: percent positive of those tested and number positive per 100,000 of population, 2012–2016, for each sex for Australia and each state or territory of residence, excluding data from the laboratory that provided positive data only. F = female, M = male, U = unknown. ACT = Australian Capital Territory; NSW = New South Wales; NT = Northern Territory; QLD = Queensland; SA = South Australia; TAS = Tasmania; VIC = Victoria; WA = Western Australia.
(DOCX)

S7 Table *Strongyloides* serology: percent positive of those tested and number positive per 100,000 of population, 2012–2016, for Australia and each state and territory by age group in years, excluding data from the laboratory that provided positive data only. ACT = Australian Capital Territory; NSW = New South Wales; NT = Northern Territory; QLD = Queensland; SA = South Australia; TAS = Tasmania; VIC = Victoria; WA = Western Australia.
(DOCX)
