# Supplementary figures and images for: Seropositivity and geographical distribution of Strongyloides stercoralis in Australia: A study of pathology laboratory data from 2012–2016
Source: PLoS Negl Trop Dis. 2021 Mar 9;15(3):e0009160. doi: 10.1371/journal.pntd.0009160 (PMC7978363; doi:10.1371/journal.pntd.0009160)

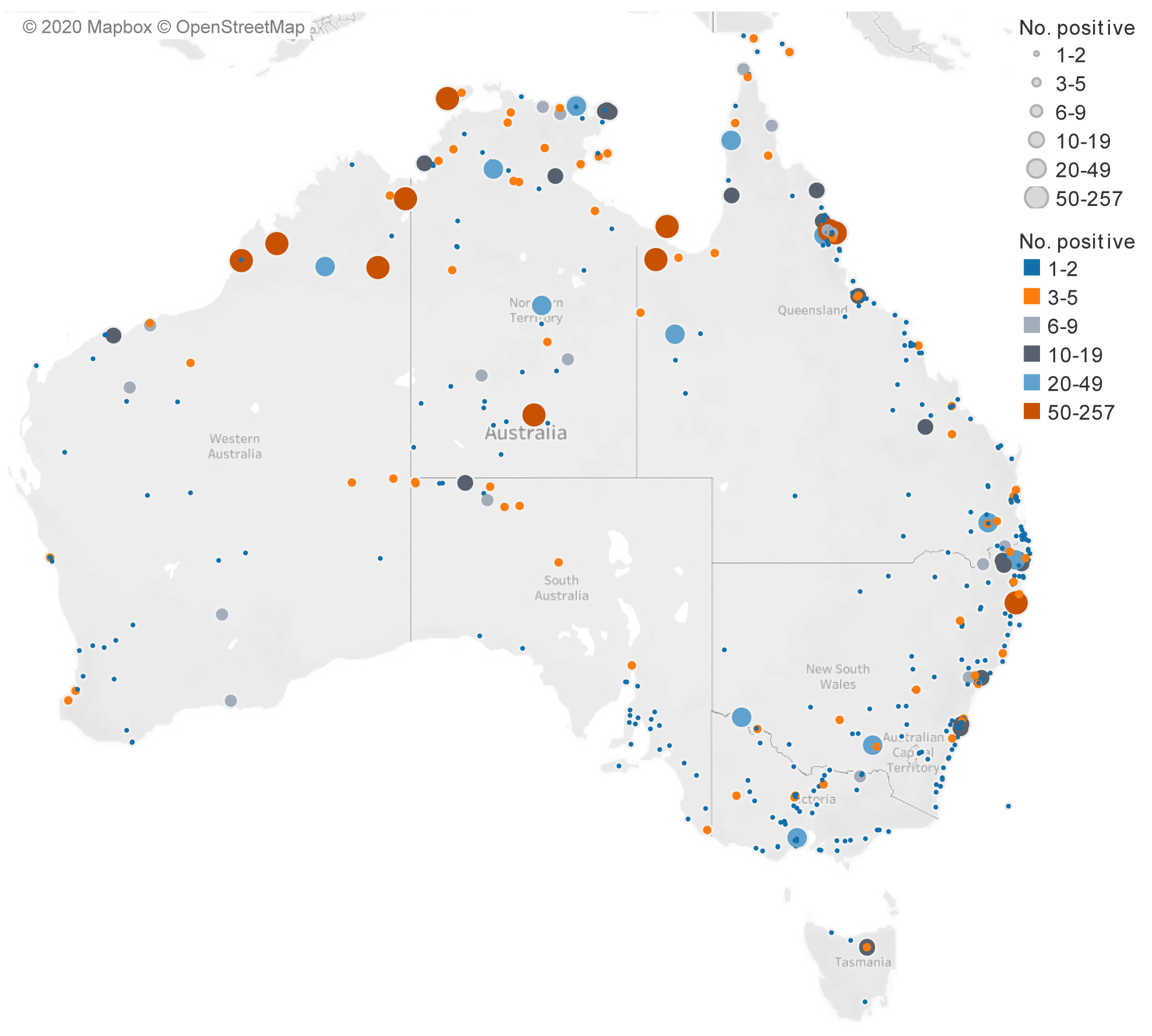

Supplement: S1 Fig — 1This map was created using our data, Tableau software and a Mapbox base map. 2The ACT and greater capital cities have been omitted. The ranges of numbers positive are shown by colour and size of the dots. ACT = Australian Capital Territory; NSW = New South Wales; NT = Northern Territory; QLD = Queensland; SA = South Australia; TAS = Tasmania; VIC = Victoria; WA = Western Australia. (TIF) [file pntd.0009160.s001.tif]

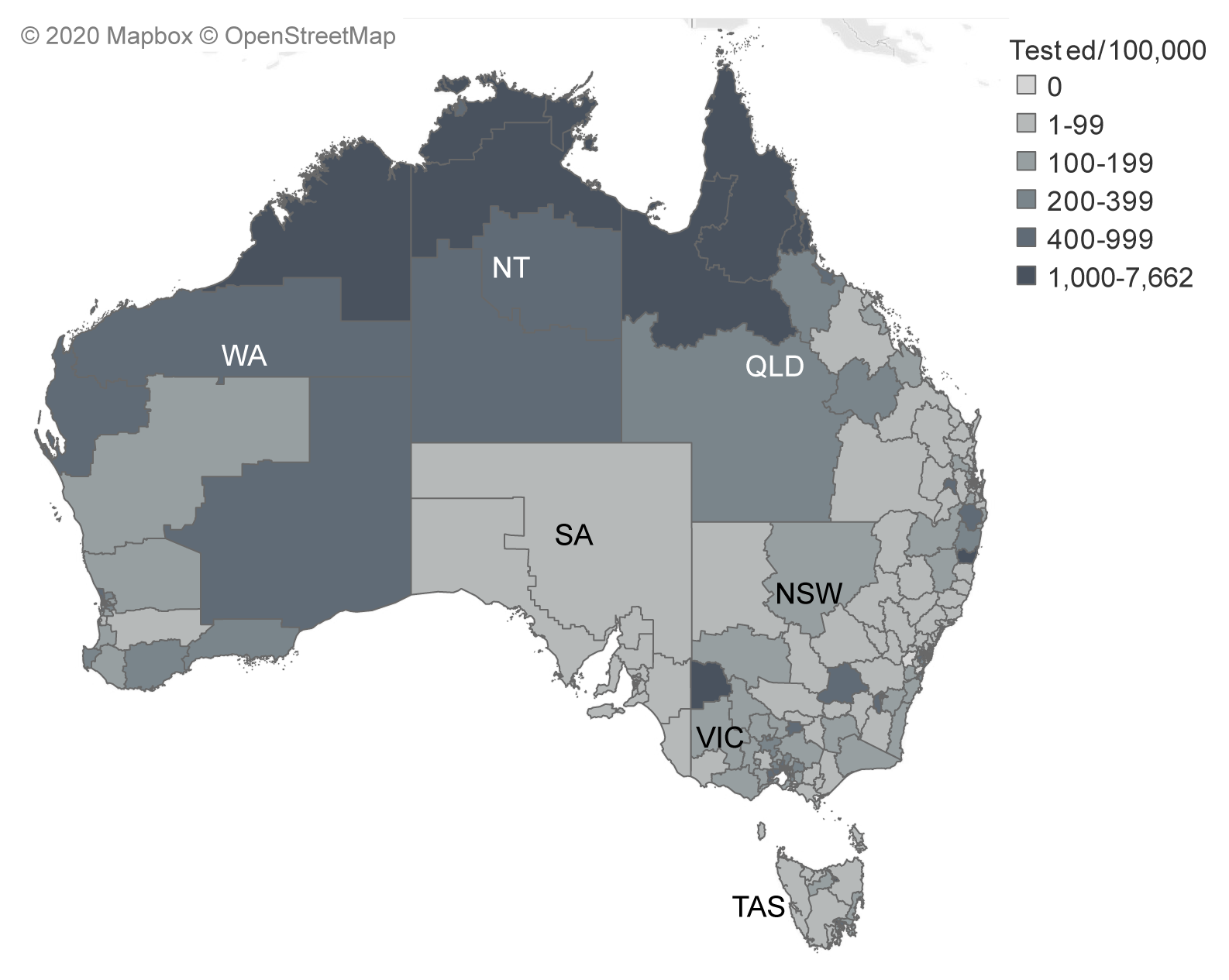

Supplement: S2 Fig — 1This map was created using our data, Tableau software, an ABS shapefile and a Mapbox base map.2This accounts for the low number of tests in South Australia. ACT = Australian Capital Territory; NSW = New South Wales; NT = Northern Territory; QLD = Queensland; SA = South Australia; TAS = Tasmania; VIC = Victoria; WA = Western Australia. (TIF) [file pntd.0009160.s002.tif]

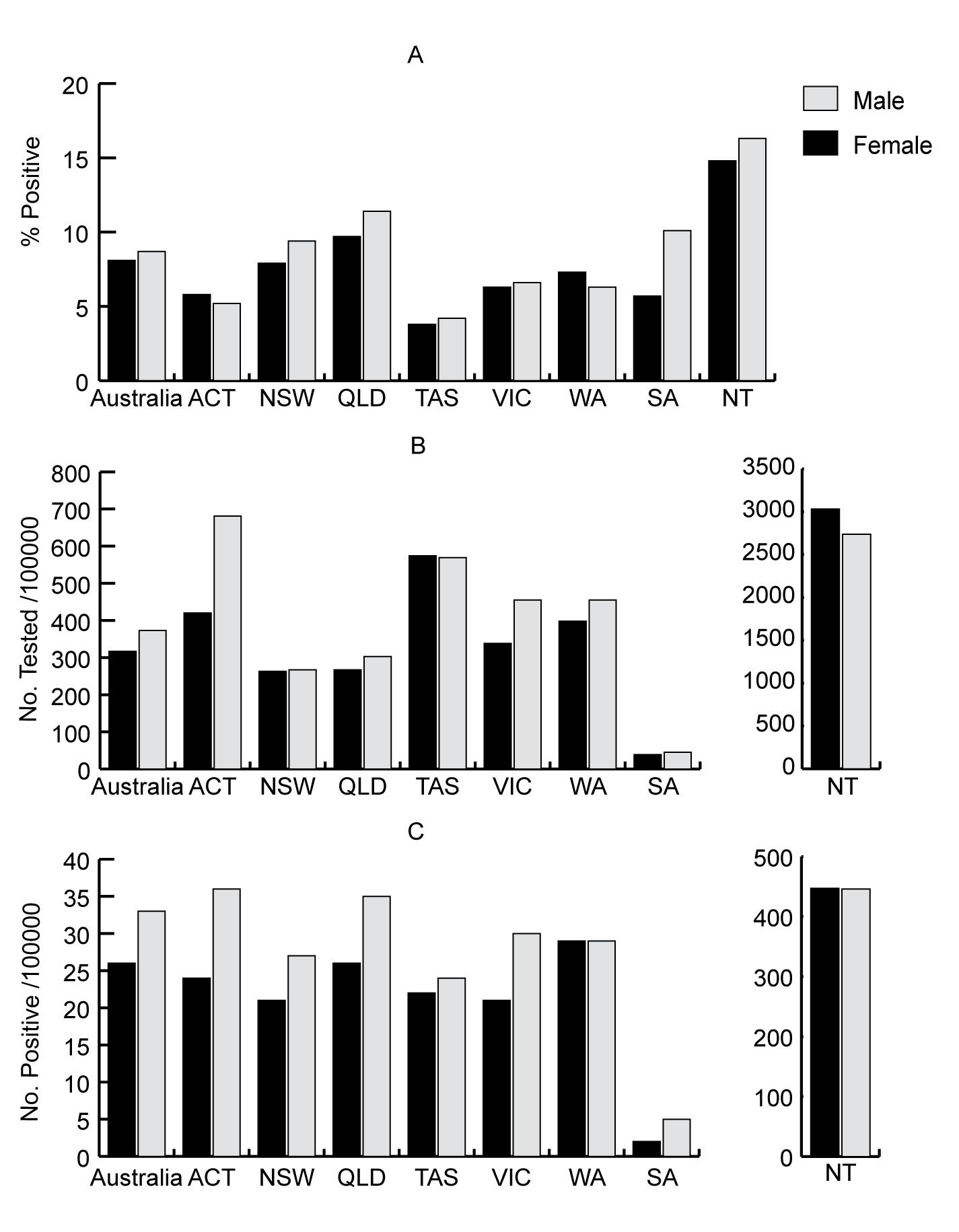

Supplement: S3 Fig — A. Percent positive of those tested. B. Number tested per 100,000 of population. C. Number positive per 100,000 of population. 1The values for SA in B and C are considerably underestimated because of the exclusion of data from the laboratory in SA. ACT = Australian Capital Territory; NSW = New South Wales; NT = Northern Territory; QLD = Queensland; SA = South Australia; TAS = Tasmania; VIC = Victoria; WA = Western Australia. (TIF) [file pntd.0009160.s003.tif]

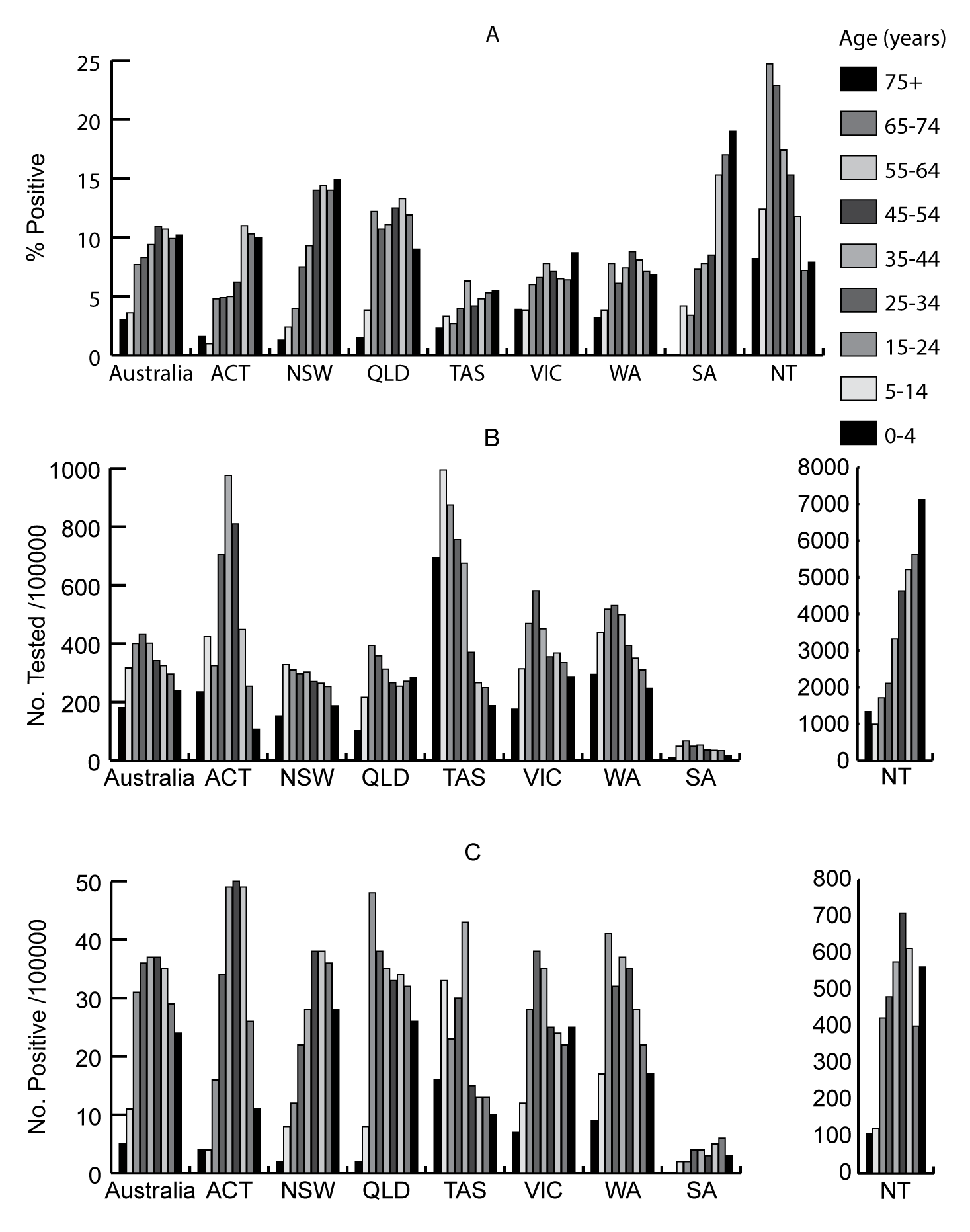

Supplement: S4 Fig — A. Percent positive in each age group. B. Number of people tested per 100,000 of population in each age group and each state. C. Number of people positive per 100,000 of population for each age group. 1The values for SA in B and C are considerably underestimated because of the exclusion of data from the laboratory in SA. ACT = Australian Capital Territory; NSW = New South Wales; NT = Northern Territory; QLD = Queensland; SA = South Australia; TAS = Tasmania; VIC = Victoria; WA = Western Australia. (TIF) [file pntd.0009160.s004.tif]
