# Supplementary material for: Seropositivity and geographical distribution of Strongyloides stercoralis in Australia: A study of pathology laboratory data from 2012–2016
Source: PLoS Negl Trop Dis. 2021 Mar 9;15(3):e0009160. doi: 10.1371/journal.pntd.0009160 (PMC7978363; doi:10.1371/journal.pntd.0009160)
Supplement: S1 Table — 1Ratio of the optical density of the test/optical density of the weak positive control. 2OD = optical density of the test solution. 3The in-house S. ratti assay at the WA laboratory was replaced by IVD ELISA during 2012. NSW = New South Wales; QLD = Queensland; SA = South Australia; VIC = Victoria; WA = Western Australia; P1 Private laboratory 1. (P2 did not contribute data to the study). (DOCX) [file pntd.0009160.s005.docx]

| Laboratory | ELISA test | Antigen | Unit | Negative | Equivocal | Positive |
| --- | --- | --- | --- | --- | --- | --- |
|  |  |  |  |  |  |  |
| SA | Bordier | *S. ratti* | ratio^1^ | <0.9 | 0.9-1.1 | >1.1 |
| QLD | Bordier | *S. ratti* | ratio^1^ | <0.9 | 0.9-1.1 | >1.1 |
| QLD P1 | Bordier | *S. ratti* | ratio^1^ | <0.9 | 0.9-1.1 | >1.1 |
|  |  |  |  |  |  |  |
| NSW | In-house | *S. ratti* | ratio^1^ | <0.8 | 0.8-1.2 | >1.2 |
|  |  |  |  |  |  |  |
| VIC | IVD | *S. stercoralis* | OD^2^ | <0.18 | 0.18-0.22 | >0.22 |
| WA^3^ | IVD | *S. stercoralis* | OD^2^ | <0.2 | 0.2-0.4 | >0.40 |
| WA^3^ | In-house | *S. ratti* | OD^2^ | <0.25 | 0.25-0.45 | >0.45 |
