# Supplementary material for: Seropositivity and geographical distribution of Strongyloides stercoralis in Australia: A study of pathology laboratory data from 2012–2016
Source: PLoS Negl Trop Dis. 2021 Mar 9;15(3):e0009160. doi: 10.1371/journal.pntd.0009160 (PMC7978363; doi:10.1371/journal.pntd.0009160)
Supplement: S2 Table — CI = confidence interval, ACT = Australian Capital Territory; NSW = New South Wales; NT = Northern Territory; QLD = Queensland; TAS = Tasmania; VIC = Victoria; WA = Western Australia; SA = South Australia. (DOCX) [file pntd.0009160.s006.docx]

| **State/Territory of residence** | **No. of people positive** | **Average annualized population** | **No. positive /100000 (95% CI)** |
| --- | --- | --- | --- |
| ACT | 117 | 389502 | 30 (25, 36) |
| NSW | 1815 | 7513103 | 24 (23, 25) |
| NT | 1184 | 242180 | 489 (462, 517) |
| QLD | 1431 | 4712802 | 30 (29, 32) |
| TAS | 117 | 514041 | 23 (19, 27) |
| VIC | 1526 | 5902834 | 26 (25, 27) |
| WA | 731 | 2505342 | 29 (27, 31) |
| SA | 576 | 1685734 | 34 (31, 37) |
| Australia | 7497 | 23465538 | 32 (31, 33) |
