# Supplementary material for: Seropositivity and geographical distribution of Strongyloides stercoralis in Australia: A study of pathology laboratory data from 2012–2016
Source: PLoS Negl Trop Dis. 2021 Mar 9;15(3):e0009160. doi: 10.1371/journal.pntd.0009160 (PMC7978363; doi:10.1371/journal.pntd.0009160)
Supplement: S3 Table — ACT = Australian Capital Territory; NSW = New South Wales; NT = Northern Territory; QLD = Queensland; SA = South Australia; TAS = Tasmania; VIC = Victoria; WA = Western Australia. (DOCX) [file pntd.0009160.s007.docx]

| **State /Territory** | **SA3 code 2011** | **SA3 name 2011** | **No. positive** | **Average**  **annualized population** | **No. positive /100,000** |
| --- | --- | --- | --- | --- | --- |
| ACT | 80101 | BELCONNEN | 23 | 96957 | 24 |
| ACT | 80102 | COTTER - NAMADGI | 0 | 2419 | 0 |
| ACT | 80103 | FYSHWICK - PIALLIGO - HUME | 1 | 1576 | 63 |
| ACT | 80104 | GUNGAHLIN | 14 | 63402 | 22 |
| ACT | 80105 | NORTH CANBERRA | 40 | 52744 | 76 |
| ACT | 80106 | SOUTH CANBERRA | 18 | 26574 | 68 |
| ACT | 80107 | TUGGERANONG | 10 | 87343 | 11 |
| ACT | 80108 | WESTON CREEK | 5 | 23548 | 21 |
| ACT | 80109 | WODEN | 6 | 34940 | 17 |
| NSW | 10101 | GOULBURN - YASS | 2 | 71721 | 3 |
| NSW | 10102 | QUEANBEYAN | 5 | 57833 | 9 |
| NSW | 10103 | SNOWY MOUNTAINS | 0 | 19644 | 0 |
| NSW | 10104 | SOUTH COAST | 9 | 71265 | 13 |
| NSW | 10201 | GOSFORD | 7 | 171858 | 4 |
| NSW | 10202 | WYONG | 5 | 158774 | 3 |
| NSW | 10301 | BATHURST | 0 | 46525 | 0 |
| NSW | 10302 | LACHLAN VALLEY | 3 | 56469 | 5 |
| NSW | 10303 | LITHGOW - MUDGEE | 1 | 46755 | 2 |
| NSW | 10304 | ORANGE | 5 | 57804 | 9 |
| NSW | 10401 | CLARENCE VALLEY | 15 | 51074 | 29 |
| NSW | 10402 | COFFS HARBOUR | 80 | 86408 | 93 |
| NSW | 10501 | BOURKE - COBAR - COONAMBLE | 5 | 25583 | 20 |
| NSW | 10502 | BROKEN HILL AND FAR WEST | 2 | 21240 | 9 |
| NSW | 10503 | DUBBO | 4 | 70373 | 6 |
| NSW | 10601 | LOWER HUNTER | 6 | 87678 | 7 |
| NSW | 10602 | MAITLAND | 0 | 72338 | 0 |
| NSW | 10603 | PORT STEPHENS | 1 | 71786 | 1 |
| NSW | 10604 | UPPER HUNTER | 1 | 30861 | 3 |
| NSW | 10701 | DAPTO - PORT KEMBLA | 16 | 76536 | 21 |
| NSW | 10702 | ILLAWARRA CATCHMENT RESERVE | 0 | 8 | 0 |
| NSW | 10703 | KIAMA - SHELLHARBOUR | 20 | 90107 | 22 |
| NSW | 10704 | WOLLONGONG | 47 | 130060 | 36 |
| NSW | 10801 | GREAT LAKES | 2 | 31427 | 6 |
| NSW | 10802 | KEMPSEY - NAMBUCCA | 5 | 48836 | 10 |
| NSW | 10804 | PORT MACQUARIE | 1 | 78111 | 1 |
| NSW | 10805 | TAREE - GLOUCESTER | 4 | 54004 | 7 |
| NSW | 10901 | ALBURY | 3 | 61104 | 5 |
| NSW | 10902 | LOWER MURRAY | 2 | 12787 | 16 |
| NSW | 10903 | UPPER MURRAY EXC. ALBURY | 1 | 42290 | 2 |
| NSW | 11001 | ARMIDALE | 2 | 37838 | 5 |
| NSW | 11002 | INVERELL - TENTERFIELD | 13 | 38880 | 33 |
| NSW | 11003 | MOREE - NARRABRI | 2 | 26610 | 8 |
| NSW | 11004 | TAMWORTH - GUNNEDAH | 7 | 81145 | 9 |
| NSW | 11101 | LAKE MACQUARIE - EAST | 1 | 122690 | 1 |
| NSW | 11102 | LAKE MACQUARIE - WEST | 7 | 75184 | 9 |
| NSW | 11103 | NEWCASTLE | 46 | 166744 | 28 |
| NSW | 11201 | RICHMOND VALLEY - COASTAL | 11 | 79217 | 14 |
| NSW | 11202 | RICHMOND VALLEY - HINTERLAND | 87 | 71664 | 121 |
| NSW | 11203 | TWEED VALLEY | 1 | 91327 | 1 |
| NSW | 11301 | GRIFFITH - MURRUMBIDGEE (WEST) | 7 | 48988 | 14 |
| NSW | 11302 | TUMUT - TUMBARUMBA | 0 | 14720 | 0 |
| NSW | 11303 | WAGGA WAGGA | 27 | 94045 | 29 |
| NSW | 11401 | SHOALHAVEN | 9 | 99240 | 9 |
| NSW | 11402 | SOUTHERN HIGHLANDS | 4 | 47603 | 8 |
| NSW | 11501 | BAULKHAM HILLS | 11 | 144784 | 8 |
| NSW | 11502 | DURAL - WISEMANS FERRY | 1 | 26737 | 4 |
| NSW | 11503 | HAWKESBURY | 0 | 25034 | 0 |
| NSW | 11504 | ROUSE HILL - MCGRATHS HILL | 3 | 31320 | 10 |
| NSW | 11601 | BLACKTOWN | 83 | 135079 | 61 |
| NSW | 11602 | BLACKTOWN - NORTH | 21 | 88036 | 24 |
| NSW | 11603 | MOUNT DRUITT | 91 | 111926 | 81 |
| NSW | 11701 | BOTANY | 5 | 44999 | 11 |
| NSW | 11702 | MARRICKVILLE - SYDENHAM - PETERSHAM | 12 | 55883 | 21 |
| NSW | 11703 | SYDNEY INNER CITY | 37 | 211121 | 18 |
| NSW | 11801 | EASTERN SUBURBS - NORTH | 13 | 133491 | 10 |
| NSW | 11802 | EASTERN SUBURBS - SOUTH | 19 | 144580 | 13 |
| NSW | 11901 | BANKSTOWN | 41 | 173516 | 24 |
| NSW | 11902 | CANTERBURY | 53 | 138165 | 38 |
| NSW | 11903 | HURSTVILLE | 13 | 128389 | 10 |
| NSW | 11904 | KOGARAH - ROCKDALE | 12 | 139240 | 9 |
| NSW | 12001 | CANADA BAY | 8 | 85598 | 9 |
| NSW | 12002 | LEICHHARDT | 4 | 57574 | 7 |
| NSW | 12003 | STRATHFIELD - BURWOOD - ASHFIELD | 25 | 153331 | 16 |
| NSW | 12101 | CHATSWOOD - LANE COVE | 10 | 111855 | 9 |
| NSW | 12102 | HORNSBY | 8 | 81628 | 10 |
| NSW | 12103 | KU-RING-GAI | 3 | 119935 | 3 |
| NSW | 12104 | NORTH SYDNEY - MOSMAN | 12 | 97772 | 12 |
| NSW | 12201 | MANLY | 3 | 44003 | 7 |
| NSW | 12202 | PITTWATER | 6 | 62071 | 10 |
| NSW | 12203 | WARRINGAH | 26 | 153710 | 17 |
| NSW | 12301 | CAMDEN | 5 | 59476 | 8 |
| NSW | 12302 | CAMPBELLTOWN (NSW) | 53 | 157985 | 34 |
| NSW | 12303 | WOLLONDILLY | 4 | 40148 | 10 |
| NSW | 12401 | BLUE MOUNTAINS | 5 | 78547 | 6 |
| NSW | 12402 | BLUE MOUNTAINS - SOUTH | 0 | 5 | 0 |
| NSW | 12403 | PENRITH | 20 | 137160 | 15 |
| NSW | 12404 | RICHMOND - WINDSOR | 0 | 37333 | 0 |
| NSW | 12405 | ST MARYS | 12 | 54918 | 22 |
| NSW | 12501 | AUBURN | 30 | 87228 | 34 |
| NSW | 12502 | CARLINGFORD | 7 | 65920 | 11 |
| NSW | 12503 | MERRYLANDS - GUILDFORD | 60 | 151621 | 40 |
| NSW | 12504 | PARRAMATTA | 57 | 140013 | 41 |
| NSW | 12601 | PENNANT HILLS - EPPING | 5 | 47349 | 11 |
| NSW | 12602 | RYDE - HUNTERS HILL | 18 | 134635 | 13 |
| NSW | 12701 | BRINGELLY - GREEN VALLEY | 85 | 95217 | 89 |
| NSW | 12702 | FAIRFIELD | 355 | 188720 | 188 |
| NSW | 12703 | LIVERPOOL | 92 | 116250 | 79 |
| NSW | 12801 | CRONULLA - MIRANDA - CARINGBAH | 6 | 112420 | 5 |
| NSW | 12802 | SUTHERLAND - MENAI - HEATHCOTE | 5 | 110849 | 5 |
| NSW | 90103 | JERVIS BAY | 0 | 377 | 0 |
| NT | 70101 | DARWIN CITY | 78 | 27176 | 287 |
| NT | 70102 | DARWIN SUBURBS | 647 | 57268 | 1130 |
| NT | 70103 | LITCHFIELD | 5 | 22942 | 22 |
| NT | 70104 | PALMERSTON | 12 | 33566 | 36 |
| NT | 70201 | ALICE SPRINGS | 105 | 40332 | 260 |
| NT | 70202 | BARKLY | 29 | 6361 | 456 |
| NT | 70203 | DALY - TIWI - WEST ARNHEM | 136 | 18213 | 747 |
| NT | 70204 | EAST ARNHEM | 113 | 15516 | 728 |
| NT | 70205 | KATHERINE | 59 | 20806 | 284 |
| QLD | 30101 | CAPALABA | 7 | 73716 | 9 |
| QLD | 30102 | CLEVELAND - STRADBROKE | 5 | 83257 | 6 |
| QLD | 30103 | WYNNUM - MANLY | 7 | 70282 | 10 |
| QLD | 30201 | BALD HILLS - EVERTON PARK | 3 | 41399 | 7 |
| QLD | 30202 | CHERMSIDE | 18 | 70602 | 25 |
| QLD | 30203 | NUNDAH | 10 | 38686 | 26 |
| QLD | 30204 | SANDGATE | 16 | 57963 | 28 |
| QLD | 30301 | CARINDALE | 2 | 51021 | 4 |
| QLD | 30302 | HOLLAND PARK - YERONGA | 29 | 72482 | 40 |
| QLD | 30303 | MT GRAVATT | 20 | 72048 | 28 |
| QLD | 30304 | NATHAN | 13 | 39715 | 33 |
| QLD | 30305 | ROCKLEA - ACACIA RIDGE | 20 | 59736 | 33 |
| QLD | 30306 | SUNNYBANK | 10 | 51080 | 20 |
| QLD | 30401 | CENTENARY | 2 | 34116 | 6 |
| QLD | 30402 | KENMORE - BROOKFIELD - MOGGILL | 4 | 46686 | 9 |
| QLD | 30403 | SHERWOOD - INDOOROOPILLY | 7 | 52137 | 13 |
| QLD | 30404 | THE GAP - ENOGGERA | 7 | 51132 | 14 |
| QLD | 30501 | BRISBANE INNER | 24 | 67682 | 35 |
| QLD | 30502 | BRISBANE INNER - EAST | 8 | 41635 | 19 |
| QLD | 30503 | BRISBANE INNER - NORTH | 10 | 86049 | 12 |
| QLD | 30504 | BRISBANE INNER - WEST | 5 | 58068 | 9 |
| QLD | 30601 | CAIRNS - NORTH | 17 | 52354 | 32 |
| QLD | 30602 | CAIRNS - SOUTH | 73 | 102308 | 71 |
| QLD | 30603 | INNISFAIL - CASSOWARY COAST | 261 | 35251 | 740 |
| QLD | 30604 | PORT DOUGLAS - DAINTREE | 17 | 11631 | 146 |
| QLD | 30605 | TABLELANDS (EAST) - KURANDA | 106 | 40580 | 261 |
| QLD | 30701 | DARLING DOWNS (WEST) - MARANOA | 2 | 45022 | 4 |
| QLD | 30702 | DARLING DOWNS - EAST | 4 | 42565 | 9 |
| QLD | 30703 | GRANITE BELT | 0 | 40375 | 0 |
| QLD | 30801 | CENTRAL HIGHLANDS (QLD) | 19 | 30380 | 63 |
| QLD | 30802 | GLADSTONE - BILOELA | 4 | 77339 | 5 |
| QLD | 30803 | ROCKHAMPTON | 9 | 117407 | 8 |
| QLD | 30901 | BROADBEACH - BURLEIGH | 0 | 63174 | 0 |
| QLD | 30902 | COOLANGATTA | 1 | 53995 | 2 |
| QLD | 30903 | GOLD COAST - NORTH | 2 | 65995 | 3 |
| QLD | 30904 | GOLD COAST HINTERLAND | 2 | 18387 | 11 |
| QLD | 30905 | MUDGEERABA - TALLEBUDGERA | 1 | 33587 | 3 |
| QLD | 30906 | NERANG | 1 | 67909 | 1 |
| QLD | 30907 | ORMEAU - OXENFORD | 2 | 112680 | 2 |
| QLD | 30908 | ROBINA | 2 | 49820 | 4 |
| QLD | 30909 | SOUTHPORT | 1 | 59380 | 2 |
| QLD | 30910 | SURFERS PARADISE | 0 | 40581 | 0 |
| QLD | 31001 | FOREST LAKE - OXLEY | 42 | 71630 | 59 |
| QLD | 31002 | IPSWICH HINTERLAND | 2 | 61906 | 3 |
| QLD | 31003 | IPSWICH INNER | 1 | 103859 | 1 |
| QLD | 31004 | SPRINGFIELD - REDBANK | 13 | 79758 | 16 |
| QLD | 31101 | BEAUDESERT | 3 | 13622 | 22 |
| QLD | 31102 | BEENLEIGH | 4 | 41201 | 10 |
| QLD | 31103 | BROWNS PLAINS | 26 | 80115 | 32 |
| QLD | 31104 | JIMBOOMBA | 0 | 43356 | 0 |
| QLD | 31105 | LOGANLEA - CARBROOK | 7 | 60029 | 12 |
| QLD | 31106 | SPRINGWOOD - KINGSTON | 103 | 79988 | 129 |
| QLD | 31201 | BOWEN BASIN - NORTH | 3 | 35841 | 8 |
| QLD | 31202 | MACKAY | 18 | 118160 | 15 |
| QLD | 31203 | WHITSUNDAY | 1 | 20968 | 5 |
| QLD | 31301 | BRIBIE - BEACHMERE | 2 | 33079 | 6 |
| QLD | 31302 | CABOOLTURE | 4 | 66361 | 6 |
| QLD | 31303 | CABOOLTURE HINTERLAND | 1 | 12911 | 8 |
| QLD | 31304 | NARANGBA - BURPENGARY | 7 | 63114 | 11 |
| QLD | 31305 | REDCLIFFE | 7 | 59955 | 12 |
| QLD | 31401 | HILLS DISTRICT | 6 | 86791 | 7 |
| QLD | 31402 | NORTH LAKES | 5 | 65174 | 8 |
| QLD | 31403 | STRATHPINE | 7 | 37919 | 18 |
| QLD | 31501 | FAR NORTH | 100 | 32718 | 306 |
| QLD | 31502 | OUTBACK - NORTH | 194 | 32950 | 589 |
| QLD | 31503 | OUTBACK - SOUTH | 2 | 19237 | 10 |
| QLD | 31601 | BUDERIM | 1 | 52724 | 2 |
| QLD | 31602 | CALOUNDRA | 3 | 78633 | 4 |
| QLD | 31603 | MAROOCHY | 2 | 56564 | 4 |
| QLD | 31604 | NAMBOUR - POMONA | 5 | 63652 | 8 |
| QLD | 31605 | NOOSA | 3 | 40545 | 7 |
| QLD | 31606 | SUNSHINE COAST HINTERLAND | 2 | 49492 | 4 |
| QLD | 31701 | TOOWOOMBA | 35 | 149935 | 23 |
| QLD | 31801 | CHARTERS TOWERS - AYR - INGHAM | 9 | 43816 | 21 |
| QLD | 31802 | TOWNSVILLE | 55 | 188107 | 29 |
| QLD | 31901 | BUNDABERG | 3 | 88649 | 3 |
| QLD | 31902 | BURNETT | 3 | 49645 | 6 |
| QLD | 31903 | GYMPIE - COOLOOLA | 0 | 48973 | 0 |
| QLD | 31904 | HERVEY BAY | 1 | 56354 | 2 |
| QLD | 31905 | MARYBOROUGH | 0 | 44857 | 0 |
| TAS | 60101 | BRIGHTON | 0 | 16809 | 0 |
| TAS | 60102 | HOBART - NORTH EAST | 12 | 52080 | 23 |
| TAS | 60103 | HOBART - NORTH WEST | 52 | 52898 | 98 |
| TAS | 60104 | HOBART - SOUTH AND WEST | 2 | 32748 | 6 |
| TAS | 60105 | HOBART INNER | 8 | 51377 | 16 |
| TAS | 60106 | SORELL - DODGES FERRY | 0 | 15641 | 0 |
| TAS | 60201 | LAUNCESTON | 39 | 82408 | 47 |
| TAS | 60202 | MEANDER VALLEY - WEST TAMAR | 1 | 22708 | 4 |
| TAS | 60203 | NORTH EAST | 0 | 37785 | 0 |
| TAS | 60301 | CENTRAL HIGHLANDS (TAS.) | 0 | 11503 | 0 |
| TAS | 60302 | HUON - BRUNY ISLAND | 1 | 19240 | 5 |
| TAS | 60303 | SOUTH EAST COAST | 0 | 6797 | 0 |
| TAS | 60401 | BURNIE - ULVERSTONE | 1 | 49038 | 2 |
| TAS | 60402 | DEVONPORT | 1 | 45004 | 2 |
| TAS | 60403 | WEST COAST | 0 | 18004 | 0 |
| VIC | 20101 | BALLARAT | 5 | 102140 | 5 |
| VIC | 20102 | CRESWICK - DAYLESFORD - BALLAN | 0 | 28134 | 0 |
| VIC | 20103 | MARYBOROUGH - PYRENEES | 1 | 24953 | 4 |
| VIC | 20201 | BENDIGO | 16 | 92439 | 17 |
| VIC | 20202 | HEATHCOTE - CASTLEMAINE - KYNETON | 4 | 46327 | 9 |
| VIC | 20203 | LODDON - ELMORE | 0 | 11402 | 0 |
| VIC | 20301 | BARWON - WEST | 0 | 18357 | 0 |
| VIC | 20302 | GEELONG | 39 | 186033 | 21 |
| VIC | 20303 | SURF COAST - BELLARINE PENINSULA | 3 | 68600 | 4 |
| VIC | 20401 | UPPER GOULBURN VALLEY | 3 | 52875 | 6 |
| VIC | 20402 | WANGARATTA - BENALLA | 0 | 45732 | 0 |
| VIC | 20403 | WODONGA - ALPINE | 11 | 69157 | 16 |
| VIC | 20501 | BAW BAW | 1 | 46932 | 2 |
| VIC | 20502 | GIPPSLAND - EAST | 5 | 44491 | 11 |
| VIC | 20503 | GIPPSLAND - SOUTH WEST | 3 | 60709 | 5 |
| VIC | 20504 | LATROBE VALLEY | 2 | 74058 | 3 |
| VIC | 20505 | WELLINGTON | 1 | 42849 | 2 |
| VIC | 20601 | BRUNSWICK - COBURG | 19 | 88073 | 22 |
| VIC | 20602 | DAREBIN - SOUTH | 11 | 53497 | 21 |
| VIC | 20603 | ESSENDON | 20 | 67393 | 30 |
| VIC | 20604 | MELBOURNE CITY | 107 | 127445 | 84 |
| VIC | 20605 | PORT PHILLIP | 17 | 104313 | 16 |
| VIC | 20606 | STONNINGTON - WEST | 7 | 63218 | 11 |
| VIC | 20607 | YARRA | 34 | 87872 | 39 |
| VIC | 20701 | BOROONDARA | 22 | 172731 | 13 |
| VIC | 20702 | MANNINGHAM - WEST | 8 | 92864 | 9 |
| VIC | 20703 | WHITEHORSE - WEST | 10 | 103401 | 10 |
| VIC | 20801 | BAYSIDE | 8 | 99840 | 8 |
| VIC | 20802 | GLEN EIRA | 18 | 151484 | 12 |
| VIC | 20803 | KINGSTON | 12 | 118590 | 10 |
| VIC | 20804 | STONNINGTON - EAST | 1 | 42746 | 2 |
| VIC | 20901 | BANYULE | 27 | 125308 | 22 |
| VIC | 20902 | DAREBIN - NORTH | 31 | 96276 | 32 |
| VIC | 20903 | NILLUMBIK - KINGLAKE | 9 | 67343 | 13 |
| VIC | 20904 | WHITTLESEA - WALLAN | 31 | 203102 | 15 |
| VIC | 21001 | KEILOR | 8 | 59407 | 13 |
| VIC | 21002 | MACEDON RANGES | 1 | 29261 | 3 |
| VIC | 21003 | MORELAND - NORTH | 21 | 75416 | 28 |
| VIC | 21004 | SUNBURY | 3 | 39479 | 8 |
| VIC | 21005 | TULLAMARINE - BROADMEADOWS | 55 | 154835 | 36 |
| VIC | 21101 | KNOX | 14 | 157693 | 9 |
| VIC | 21102 | MANNINGHAM - EAST | 3 | 26970 | 11 |
| VIC | 21103 | MAROONDAH | 124 | 111051 | 112 |
| VIC | 21104 | WHITEHORSE - EAST | 11 | 61820 | 18 |
| VIC | 21105 | YARRA RANGES | 50 | 151436 | 33 |
| VIC | 21201 | CARDINIA | 11 | 88761 | 12 |
| VIC | 21202 | CASEY - NORTH | 53 | 133622 | 40 |
| VIC | 21203 | CASEY - SOUTH | 20 | 155881 | 13 |
| VIC | 21204 | DANDENONG | 251 | 188250 | 133 |
| VIC | 21205 | MONASH | 20 | 178534 | 11 |
| VIC | 21301 | BRIMBANK | 87 | 189882 | 46 |
| VIC | 21302 | HOBSONS BAY | 12 | 85744 | 14 |
| VIC | 21303 | MARIBYRNONG | 31 | 82230 | 38 |
| VIC | 21304 | MELTON - BACCHUS MARSH | 15 | 146977 | 10 |
| VIC | 21305 | WYNDHAM | 175 | 207204 | 84 |
| VIC | 21401 | FRANKSTON | 5 | 136004 | 4 |
| VIC | 21402 | MORNINGTON PENINSULA | 17 | 156512 | 11 |
| VIC | 21501 | GRAMPIANS | 6 | 59730 | 10 |
| VIC | 21502 | MILDURA | 26 | 53636 | 48 |
| VIC | 21503 | MURRAY RIVER - SWAN HILL | 6 | 37822 | 16 |
| VIC | 21601 | CAMPASPE | 2 | 37482 | 5 |
| VIC | 21602 | MOIRA | 2 | 29091 | 7 |
| VIC | 21603 | SHEPPARTON | 7 | 63664 | 11 |
| VIC | 21701 | GLENELG - SOUTHERN GRAMPIANS | 0 | 36106 | 0 |
| VIC | 21702 | WARRNAMBOOL - OTWAY RANGES | 4 | 87648 | 5 |
| WA | 50101 | AUGUSTA - MARGARET RIVER - BUSSELTON | 3 | 49552 | 6 |
| WA | 50102 | BUNBURY | 7 | 102917 | 7 |
| WA | 50103 | MANJIMUP | 0 | 22964 | 0 |
| WA | 50201 | MANDURAH | 3 | 96075 | 3 |
| WA | 50301 | COTTESLOE - CLAREMONT | 6 | 71045 | 8 |
| WA | 50302 | PERTH CITY | 8 | 105418 | 8 |
| WA | 50401 | BAYSWATER - BASSENDEAN | 12 | 83881 | 14 |
| WA | 50402 | MUNDARING | 8 | 43462 | 18 |
| WA | 50403 | SWAN | 26 | 123702 | 21 |
| WA | 50501 | JOONDALUP | 6 | 163325 | 4 |
| WA | 50502 | STIRLING | 53 | 199687 | 27 |
| WA | 50503 | WANNEROO | 36 | 182025 | 20 |
| WA | 50601 | ARMADALE | 9 | 75638 | 12 |
| WA | 50602 | BELMONT - VICTORIA PARK | 9 | 72564 | 12 |
| WA | 50603 | CANNING | 16 | 97011 | 16 |
| WA | 50604 | GOSNELLS | 23 | 120015 | 19 |
| WA | 50605 | KALAMUNDA | 10 | 58781 | 17 |
| WA | 50606 | SERPENTINE - JARRAHDALE | 0 | 23851 | 0 |
| WA | 50607 | SOUTH PERTH | 3 | 44190 | 7 |
| WA | 50701 | COCKBURN | 10 | 101863 | 10 |
| WA | 50702 | FREMANTLE | 4 | 38127 | 10 |
| WA | 50703 | KWINANA | 6 | 36410 | 16 |
| WA | 50704 | MELVILLE | 5 | 106350 | 5 |
| WA | 50705 | ROCKINGHAM | 4 | 122351 | 3 |
| WA | 50801 | ESPERANCE | 6 | 16495 | 36 |
| WA | 50802 | GASCOYNE | 3 | 9834 | 31 |
| WA | 50803 | GOLDFIELDS | 19 | 42811 | 44 |
| WA | 50804 | KIMBERLEY | 374 | 37542 | 996 |
| WA | 50805 | MID WEST | 10 | 56302 | 18 |
| WA | 50806 | PILBARA | 43 | 63488 | 68 |
| WA | 50901 | ALBANY | 3 | 59695 | 5 |
| WA | 50902 | WHEAT BELT - NORTH | 5 | 56798 | 9 |
| WA | 50903 | WHEAT BELT - SOUTH | 1 | 21177 | 5 |
| SA | 40101 | ADELAIDE CITY | 11 | 22588 | 49 |
| SA | 40102 | ADELAIDE HILLS | 1 | 71727 | 1 |
| SA | 40103 | BURNSIDE | 3 | 44714 | 7 |
| SA | 40104 | CAMPBELLTOWN (SA) | 2 | 51047 | 4 |
| SA | 40105 | NORWOOD - PAYNEHAM - ST PETERS | 8 | 36363 | 22 |
| SA | 40106 | PROSPECT - WALKERVILLE | 10 | 28559 | 35 |
| SA | 40107 | UNLEY | 5 | 38705 | 13 |
| SA | 40201 | GAWLER - TWO WELLS | 1 | 33818 | 3 |
| SA | 40202 | PLAYFORD | 77 | 87827 | 88 |
| SA | 40203 | PORT ADELAIDE - EAST | 49 | 66797 | 73 |
| SA | 40204 | SALISBURY | 186 | 135895 | 137 |
| SA | 40205 | TEA TREE GULLY | 5 | 94661 | 5 |
| SA | 40301 | HOLDFAST BAY | 3 | 34695 | 9 |
| SA | 40302 | MARION | 23 | 90116 | 26 |
| SA | 40303 | MITCHAM | 7 | 64642 | 11 |
| SA | 40304 | ONKAPARINGA | 14 | 168592 | 8 |
| SA | 40401 | CHARLES STURT | 59 | 109599 | 54 |
| SA | 40402 | PORT ADELAIDE - WEST | 20 | 59313 | 34 |
| SA | 40403 | WEST TORRENS | 9 | 61761 | 15 |
| SA | 40501 | BAROSSA | 2 | 35321 | 6 |
| SA | 40502 | LOWER NORTH | 2 | 22828 | 9 |
| SA | 40503 | MID NORTH | 1 | 27886 | 4 |
| SA | 40504 | YORKE PENINSULA | 8 | 25257 | 32 |
| SA | 40601 | EYRE PENINSULA AND SOUTH WEST | 5 | 58306 | 9 |
| SA | 40602 | OUTBACK - NORTH AND EAST | 52 | 28288 | 184 |
| SA | 40701 | FLEURIEU - KANGAROO ISLAND | 3 | 50006 | 6 |
| SA | 40702 | LIMESTONE COAST | 7 | 65926 | 11 |
| SA | 40703 | MURRAY AND MALLEE | 3 | 70495 | 4 |
| Total |  |  | 7497 | 23465538 | 32 |
