# Supplementary material for: Seropositivity and geographical distribution of Strongyloides stercoralis in Australia: A study of pathology laboratory data from 2012–2016
Source: PLoS Negl Trop Dis. 2021 Mar 9;15(3):e0009160. doi: 10.1371/journal.pntd.0009160 (PMC7978363; doi:10.1371/journal.pntd.0009160)
Supplement: S4 Table — 1This accounts for the low number of tests in South Australia. ACT = Australian Capital Territory; NSW = New South Wales; NT = Northern Territory; QLD = Queensland; SA = South Australia; TAS = Tasmania; VIC = Victoria; WA = Western Australia. (DOCX) [file pntd.0009160.s008.docx]

| **State /Territory** | **SA3 code 2011** | **SA3 name 2011** | **No. tested** | **No. positive** | **% Positive** | **Average**  **annualized population** | **No. tested /100,000** | **No. positive /100,000** |
| --- | --- | --- | --- | --- | --- | --- | --- | --- |
| ACT | 80101 | BELCONNEN | 362 | 23 | 6.3 | 96957 | 373 | 24 |
| ACT | 80102 | COTTER - NAMADGI | 22 | 0 | 0.0 | 2419 | 910 | 0 |
| ACT | 80104 | GUNGAHLIN | 161 | 14 | 8.7 | 63402 | 254 | 22 |
| ACT | 80105 | NORTH CANBERRA | 837 | 40 | 4.8 | 52744 | 1587 | 76 |
| ACT | 80106 | SOUTH CANBERRA | 419 | 18 | 4.3 | 26574 | 1577 | 68 |
| ACT | 80107 | TUGGERANONG | 170 | 10 | 5.9 | 87343 | 195 | 11 |
| ACT | 80108 | WESTON CREEK | 72 | 5 | 6.9 | 23548 | 306 | 21 |
| ACT | 80109 | WODEN | 87 | 6 | 6.9 | 34940 | 249 | 17 |
| NSW | 10101 | GOULBURN - YASS | 43 | 2 | 4.7 | 71721 | 60 | 3 |
| NSW | 10102 | QUEANBEYAN | 66 | 5 | 7.6 | 57833 | 114 | 9 |
| NSW | 10103 | SNOWY MOUNTAINS | 16 | 0 | 0.0 | 19644 | 81 | 0 |
| NSW | 10104 | SOUTH COAST | 73 | 9 | 12.3 | 71265 | 102 | 13 |
| NSW | 10201 | GOSFORD | 57 | 7 | 12.3 | 171858 | 33 | 4 |
| NSW | 10202 | WYONG | 71 | 4 | 5.6 | 158774 | 45 | 3 |
| NSW | 10301 | BATHURST | 22 | 0 | 0.0 | 46525 | 47 | 0 |
| NSW | 10302 | LACHLAN VALLEY | 16 | 3 | 18.8 | 56469 | 28 | 5 |
| NSW | 10303 | LITHGOW - MUDGEE | 22 | 1 | 4.6 | 46755 | 47 | 2 |
| NSW | 10304 | ORANGE | 35 | 5 | 14.3 | 57804 | 61 | 9 |
| NSW | 10401 | CLARENCE VALLEY | 105 | 15 | 14.3 | 51074 | 206 | 29 |
| NSW | 10402 | COFFS HARBOUR | 1070 | 80 | 7.5 | 86408 | 1238 | 93 |
| NSW | 10501 | BOURKE - COBAR - COONAMBLE | 26 | 5 | 19.2 | 25583 | 102 | 20 |
| NSW | 10502 | BROKEN HILL AND FAR WEST | 9 | 2 | 22.2 | 21240 | 42 | 9 |
| NSW | 10503 | DUBBO | 43 | 4 | 9.3 | 70373 | 61 | 6 |
| NSW | 10601 | LOWER HUNTER | 40 | 6 | 15.0 | 87678 | 46 | 7 |
| NSW | 10602 | MAITLAND | 13 | 0 | 0.0 | 72338 | 18 | 0 |
| NSW | 10603 | PORT STEPHENS | 8 | 1 | 12.5 | 71786 | 11 | 1 |
| NSW | 10604 | UPPER HUNTER | 8 | 1 | 12.5 | 30861 | 26 | 3 |
| NSW | 10701 | DAPTO - PORT KEMBLA | 389 | 16 | 4.1 | 76536 | 508 | 21 |
| NSW | 10702 | ILLAWARRA CATCHMENT RESERVE | 0 | 0 |  | 8 | 0 | 0 |
| NSW | 10703 | KIAMA - SHELLHARBOUR | 256 | 20 | 7.8 | 90107 | 284 | 22 |
| NSW | 10704 | WOLLONGONG | 661 | 47 | 7.1 | 130060 | 508 | 36 |
| NSW | 10801 | GREAT LAKES | 6 | 2 | 33.3 | 31427 | 19 | 6 |
| NSW | 10802 | KEMPSEY - NAMBUCCA | 24 | 5 | 20.8 | 48836 | 49 | 10 |
| NSW | 10804 | PORT MACQUARIE | 15 | 0 | 0.0 | 78111 | 19 | 0 |
| NSW | 10805 | TAREE - GLOUCESTER | 8 | 4 | 50.0 | 54004 | 15 | 7 |
| NSW | 10901 | ALBURY | 44 | 3 | 6.8 | 61104 | 72 | 5 |
| NSW | 10902 | LOWER MURRAY | 22 | 2 | 9.1 | 12787 | 172 | 16 |
| NSW | 10903 | UPPER MURRAY EXC. ALBURY | 26 | 1 | 3.8 | 42290 | 61 | 2 |
| NSW | 11001 | ARMIDALE | 45 | 2 | 4.4 | 37838 | 119 | 5 |
| NSW | 11002 | INVERELL - TENTERFIELD | 68 | 13 | 19.1 | 38880 | 175 | 33 |
| NSW | 11003 | MOREE - NARRABRI | 14 | 2 | 14.3 | 26610 | 53 | 8 |
| NSW | 11004 | TAMWORTH - GUNNEDAH | 51 | 7 | 13.7 | 81145 | 63 | 9 |
| NSW | 11101 | LAKE MACQUARIE - EAST | 26 | 1 | 3.8 | 122690 | 21 | 1 |
| NSW | 11102 | LAKE MACQUARIE - WEST | 36 | 7 | 19.4 | 75184 | 48 | 9 |
| NSW | 11103 | NEWCASTLE | 863 | 46 | 5.3 | 166744 | 518 | 28 |
| NSW | 11201 | RICHMOND VALLEY - COASTAL | 111 | 11 | 9.9 | 79217 | 140 | 14 |
| NSW | 11202 | RICHMOND VALLEY - HINTERLAND | 517 | 87 | 16.8 | 71664 | 721 | 121 |
| NSW | 11203 | TWEED VALLEY | 42 | 1 | 2.4 | 91327 | 46 | 1 |
| NSW | 11301 | GRIFFITH - MURRUMBIDGEE (WEST) | 30 | 7 | 23.3 | 48988 | 61 | 14 |
| NSW | 11302 | TUMUT - TUMBARUMBA | 6 | 0 | 0.0 | 14720 | 41 | 0 |
| NSW | 11303 | WAGGA WAGGA | 430 | 27 | 6.3 | 94045 | 457 | 29 |
| NSW | 11401 | SHOALHAVEN | 120 | 9 | 7.5 | 99240 | 121 | 9 |
| NSW | 11402 | SOUTHERN HIGHLANDS | 48 | 4 | 8.3 | 47603 | 101 | 8 |
| NSW | 11501 | BAULKHAM HILLS | 225 | 11 | 4.9 | 144784 | 155 | 8 |
| NSW | 11502 | DURAL - WISEMANS FERRY | 19 | 1 | 5.3 | 26737 | 71 | 4 |
| NSW | 11503 | HAWKESBURY | 11 | 0 | 0.0 | 25034 | 44 | 0 |
| NSW | 11504 | ROUSE HILL - MCGRATHS HILL | 49 | 3 | 6.1 | 31320 | 156 | 10 |
| NSW | 11601 | BLACKTOWN | 1091 | 83 | 7.6 | 135079 | 808 | 61 |
| NSW | 11602 | BLACKTOWN - NORTH | 252 | 21 | 8.3 | 88036 | 286 | 24 |
| NSW | 11603 | MOUNT DRUITT | 1103 | 91 | 8.3 | 111926 | 985 | 81 |
| NSW | 11701 | BOTANY | 81 | 5 | 6.2 | 44999 | 180 | 11 |
| NSW | 11702 | MARRICKVILLE - SYDENHAM - PETERSHAM | 66 | 12 | 18.2 | 55883 | 118 | 21 |
| NSW | 11703 | SYDNEY INNER CITY | 391 | 37 | 9.5 | 211121 | 185 | 18 |
| NSW | 11801 | EASTERN SUBURBS - NORTH | 130 | 13 | 10.0 | 133491 | 97 | 10 |
| NSW | 11802 | EASTERN SUBURBS - SOUTH | 183 | 19 | 10.4 | 144580 | 127 | 13 |
| NSW | 11901 | BANKSTOWN | 478 | 41 | 8.6 | 173516 | 275 | 24 |
| NSW | 11902 | CANTERBURY | 530 | 53 | 10.0 | 138165 | 384 | 38 |
| NSW | 11903 | HURSTVILLE | 125 | 13 | 10.4 | 128389 | 97 | 10 |
| NSW | 11904 | KOGARAH - ROCKDALE | 155 | 12 | 7.7 | 139240 | 111 | 9 |
| NSW | 12001 | CANADA BAY | 121 | 8 | 6.6 | 85598 | 141 | 9 |
| NSW | 12002 | LEICHHARDT | 81 | 4 | 4.9 | 57574 | 141 | 7 |
| NSW | 12003 | STRATHFIELD - BURWOOD - ASHFIELD | 255 | 25 | 9.8 | 153331 | 166 | 16 |
| NSW | 12101 | CHATSWOOD - LANE COVE | 133 | 10 | 7.5 | 111855 | 119 | 9 |
| NSW | 12102 | HORNSBY | 70 | 8 | 11.4 | 81628 | 86 | 10 |
| NSW | 12103 | KU-RING-GAI | 141 | 3 | 2.1 | 119935 | 118 | 3 |
| NSW | 12104 | NORTH SYDNEY - MOSMAN | 166 | 12 | 7.2 | 97772 | 170 | 12 |
| NSW | 12201 | MANLY | 58 | 3 | 5.2 | 44003 | 132 | 7 |
| NSW | 12202 | PITTWATER | 105 | 6 | 5.7 | 62071 | 169 | 10 |
| NSW | 12203 | WARRINGAH | 400 | 26 | 6.5 | 153710 | 260 | 17 |
| NSW | 12301 | CAMDEN | 44 | 5 | 11.4 | 59476 | 74 | 8 |
| NSW | 12302 | CAMPBELLTOWN (NSW) | 371 | 53 | 14.3 | 157985 | 235 | 34 |
| NSW | 12303 | WOLLONDILLY | 39 | 4 | 10.3 | 40148 | 97 | 10 |
| NSW | 12401 | BLUE MOUNTAINS | 53 | 5 | 9.4 | 78547 | 67 | 6 |
| NSW | 12402 | BLUE MOUNTAINS - SOUTH | 0 | 0 |  | 5 | 0 | 0 |
| NSW | 12403 | PENRITH | 203 | 20 | 9.9 | 137160 | 148 | 15 |
| NSW | 12404 | RICHMOND - WINDSOR | 18 | 0 | 0.0 | 37333 | 48 | 0 |
| NSW | 12405 | ST MARYS | 191 | 12 | 6.3 | 54918 | 348 | 22 |
| NSW | 12501 | AUBURN | 462 | 30 | 6.5 | 87228 | 530 | 34 |
| NSW | 12502 | CARLINGFORD | 105 | 7 | 6.7 | 65920 | 159 | 11 |
| NSW | 12503 | MERRYLANDS - GUILDFORD | 863 | 60 | 6.9 | 151621 | 569 | 40 |
| NSW | 12504 | PARRAMATTA | 745 | 57 | 7.7 | 140013 | 532 | 41 |
| NSW | 12601 | PENNANT HILLS - EPPING | 41 | 5 | 12.2 | 47349 | 87 | 11 |
| NSW | 12602 | RYDE - HUNTERS HILL | 203 | 18 | 8.9 | 134635 | 151 | 13 |
| NSW | 12701 | BRINGELLY - GREEN VALLEY | 715 | 85 | 11.9 | 95217 | 751 | 89 |
| NSW | 12702 | FAIRFIELD | 3079 | 355 | 11.5 | 188720 | 1632 | 188 |
| NSW | 12703 | LIVERPOOL | 1416 | 92 | 6.5 | 116250 | 1218 | 79 |
| NSW | 12801 | CRONULLA - MIRANDA - CARINGBAH | 74 | 6 | 8.1 | 112420 | 66 | 5 |
| NSW | 12802 | SUTHERLAND - MENAI - HEATHCOTE | 74 | 5 | 6.8 | 110849 | 67 | 5 |
| NSW | 90103 | JERVIS BAY | 1 | 0 | 0.0 | 377 | 265 | 0 |
| NT | 70101 | DARWIN CITY | 652 | 76 | 11.7 | 27176 | 2399 | 280 |
| NT | 70102 | DARWIN SUBURBS | 4388 | 646 | 14.7 | 57268 | 7662 | 1128 |
| NT | 70103 | LITCHFIELD | 96 | 5 | 5.2 | 22942 | 418 | 22 |
| NT | 70104 | PALMERSTON | 174 | 12 | 6.9 | 33566 | 518 | 36 |
| NT | 70201 | ALICE SPRINGS | 306 | 43 | 14.1 | 40332 | 759 | 107 |
| NT | 70202 | BARKLY | 38 | 4 | 10.5 | 6361 | 597 | 63 |
| NT | 70203 | DALY - TIWI - WEST ARNHEM | 479 | 131 | 27.4 | 18213 | 2630 | 719 |
| NT | 70204 | EAST ARNHEM | 671 | 112 | 16.7 | 15516 | 4325 | 722 |
| NT | 70205 | KATHERINE | 227 | 58 | 25.5 | 20806 | 1091 | 279 |
| QLD | 30101 | CAPALABA | 118 | 7 | 5.9 | 73716 | 160 | 9 |
| QLD | 30102 | CLEVELAND - STRADBROKE | 126 | 5 | 4.0 | 83257 | 151 | 6 |
| QLD | 30103 | WYNNUM - MANLY | 116 | 7 | 6.0 | 70282 | 165 | 10 |
| QLD | 30201 | BALD HILLS - EVERTON PARK | 49 | 3 | 6.1 | 41399 | 118 | 7 |
| QLD | 30202 | CHERMSIDE | 182 | 18 | 9.9 | 70602 | 258 | 25 |
| QLD | 30203 | NUNDAH | 82 | 10 | 12.2 | 38686 | 212 | 26 |
| QLD | 30204 | SANDGATE | 153 | 16 | 10.5 | 57963 | 264 | 28 |
| QLD | 30301 | CARINDALE | 118 | 2 | 1.7 | 51021 | 231 | 4 |
| QLD | 30302 | HOLLAND PARK - YERONGA | 412 | 29 | 7.0 | 72482 | 568 | 40 |
| QLD | 30303 | MT GRAVATT | 170 | 20 | 11.8 | 72048 | 236 | 28 |
| QLD | 30304 | NATHAN | 212 | 13 | 6.1 | 39715 | 534 | 33 |
| QLD | 30305 | ROCKLEA - ACACIA RIDGE | 292 | 20 | 6.8 | 59736 | 489 | 33 |
| QLD | 30306 | SUNNYBANK | 217 | 10 | 4.6 | 51080 | 425 | 20 |
| QLD | 30401 | CENTENARY | 43 | 2 | 4.7 | 34116 | 126 | 6 |
| QLD | 30402 | KENMORE - BROOKFIELD - MOGGILL | 82 | 4 | 4.9 | 46686 | 176 | 9 |
| QLD | 30403 | SHERWOOD - INDOOROOPILLY | 139 | 7 | 5.0 | 52137 | 267 | 13 |
| QLD | 30404 | THE GAP - ENOGGERA | 72 | 7 | 9.7 | 51132 | 141 | 14 |
| QLD | 30501 | BRISBANE INNER | 362 | 24 | 6.6 | 67682 | 535 | 35 |
| QLD | 30502 | BRISBANE INNER - EAST | 142 | 8 | 5.6 | 41635 | 341 | 19 |
| QLD | 30503 | BRISBANE INNER - NORTH | 184 | 10 | 5.4 | 86049 | 214 | 12 |
| QLD | 30504 | BRISBANE INNER - WEST | 96 | 5 | 5.2 | 58068 | 165 | 9 |
| QLD | 30601 | CAIRNS - NORTH | 172 | 17 | 9.9 | 52354 | 329 | 32 |
| QLD | 30602 | CAIRNS - SOUTH | 671 | 73 | 10.9 | 102308 | 656 | 71 |
| QLD | 30603 | INNISFAIL - CASSOWARY COAST | 1237 | 261 | 21.1 | 35251 | 3509 | 740 |
| QLD | 30604 | PORT DOUGLAS - DAINTREE | 77 | 17 | 22.1 | 11631 | 662 | 146 |
| QLD | 30605 | TABLELANDS (EAST) - KURANDA | 776 | 106 | 13.7 | 40580 | 1912 | 261 |
| QLD | 30701 | DARLING DOWNS (WEST) - MARANOA | 41 | 2 | 4.9 | 45022 | 91 | 4 |
| QLD | 30702 | DARLING DOWNS - EAST | 30 | 4 | 13.3 | 42565 | 70 | 9 |
| QLD | 30703 | GRANITE BELT | 30 | 0 | 0.0 | 40375 | 74 | 0 |
| QLD | 30801 | CENTRAL HIGHLANDS (QLD) | 98 | 19 | 19.4 | 30380 | 323 | 63 |
| QLD | 30802 | GLADSTONE - BILOELA | 51 | 4 | 7.8 | 77339 | 66 | 5 |
| QLD | 30803 | ROCKHAMPTON | 118 | 9 | 7.6 | 117407 | 101 | 8 |
| QLD | 30901 | BROADBEACH - BURLEIGH | 36 | 0 | 0.0 | 63174 | 57 | 0 |
| QLD | 30902 | COOLANGATTA | 29 | 1 | 3.5 | 53995 | 54 | 2 |
| QLD | 30903 | GOLD COAST - NORTH | 45 | 2 | 4.4 | 65995 | 68 | 3 |
| QLD | 30904 | GOLD COAST HINTERLAND | 17 | 2 | 11.8 | 18387 | 92 | 11 |
| QLD | 30905 | MUDGEERABA - TALLEBUDGERA | 12 | 1 | 8.3 | 33587 | 36 | 3 |
| QLD | 30906 | NERANG | 25 | 1 | 4.0 | 67909 | 37 | 1 |
| QLD | 30907 | ORMEAU - OXENFORD | 78 | 2 | 2.6 | 112680 | 69 | 2 |
| QLD | 30908 | ROBINA | 18 | 2 | 11.1 | 49820 | 36 | 4 |
| QLD | 30909 | SOUTHPORT | 41 | 1 | 2.4 | 59380 | 69 | 2 |
| QLD | 30910 | SURFERS PARADISE | 28 | 0 | 0.0 | 40581 | 69 | 0 |
| QLD | 31001 | FOREST LAKE - OXLEY | 606 | 42 | 6.9 | 71630 | 846 | 59 |
| QLD | 31002 | IPSWICH HINTERLAND | 48 | 2 | 4.2 | 61906 | 78 | 3 |
| QLD | 31003 | IPSWICH INNER | 68 | 1 | 1.5 | 103859 | 65 | 1 |
| QLD | 31004 | SPRINGFIELD - REDBANK | 144 | 13 | 9.0 | 79758 | 181 | 16 |
| QLD | 31101 | BEAUDESERT | 14 | 3 | 21.4 | 13622 | 103 | 22 |
| QLD | 31102 | BEENLEIGH | 46 | 4 | 8.7 | 41201 | 112 | 10 |
| QLD | 31103 | BROWNS PLAINS | 225 | 26 | 11.6 | 80115 | 281 | 32 |
| QLD | 31104 | JIMBOOMBA | 18 | 0 | 0.0 | 43356 | 42 | 0 |
| QLD | 31105 | LOGANLEA - CARBROOK | 129 | 7 | 5.4 | 60029 | 215 | 12 |
| QLD | 31106 | SPRINGWOOD - KINGSTON | 1335 | 103 | 7.7 | 79988 | 1669 | 129 |
| QLD | 31201 | BOWEN BASIN - NORTH | 35 | 3 | 8.6 | 35841 | 98 | 8 |
| QLD | 31202 | MACKAY | 172 | 18 | 10.5 | 118160 | 146 | 15 |
| QLD | 31203 | WHITSUNDAY | 20 | 1 | 5.0 | 20968 | 95 | 5 |
| QLD | 31301 | BRIBIE - BEACHMERE | 27 | 2 | 7.4 | 33079 | 82 | 6 |
| QLD | 31302 | CABOOLTURE | 70 | 4 | 5.7 | 66361 | 105 | 6 |
| QLD | 31303 | CABOOLTURE HINTERLAND | 13 | 1 | 7.7 | 12911 | 101 | 8 |
| QLD | 31304 | NARANGBA - BURPENGARY | 69 | 7 | 10.1 | 63114 | 109 | 11 |
| QLD | 31305 | REDCLIFFE | 87 | 7 | 8.1 | 59955 | 145 | 12 |
| QLD | 31401 | HILLS DISTRICT | 92 | 6 | 6.5 | 86791 | 106 | 7 |
| QLD | 31402 | NORTH LAKES | 63 | 5 | 7.9 | 65174 | 97 | 8 |
| QLD | 31403 | STRATHPINE | 57 | 7 | 12.3 | 37919 | 150 | 18 |
| QLD | 31501 | FAR NORTH | 578 | 100 | 17.3 | 32718 | 1767 | 306 |
| QLD | 31502 | OUTBACK - NORTH | 480 | 194 | 40.4 | 32950 | 1457 | 589 |
| QLD | 31503 | OUTBACK - SOUTH | 41 | 2 | 4.9 | 19237 | 213 | 10 |
| QLD | 31601 | BUDERIM | 31 | 1 | 3.2 | 52724 | 59 | 2 |
| QLD | 31602 | CALOUNDRA | 43 | 3 | 7.0 | 78633 | 55 | 4 |
| QLD | 31603 | MAROOCHY | 38 | 2 | 5.3 | 56564 | 67 | 4 |
| QLD | 31604 | NAMBOUR - POMONA | 48 | 5 | 10.4 | 63652 | 75 | 8 |
| QLD | 31605 | NOOSA | 26 | 3 | 11.5 | 40545 | 64 | 7 |
| QLD | 31606 | SUNSHINE COAST HINTERLAND | 39 | 2 | 5.1 | 49492 | 79 | 4 |
| QLD | 31701 | TOOWOOMBA | 728 | 35 | 4.8 | 149935 | 486 | 23 |
| QLD | 31801 | CHARTERS TOWERS - AYR - INGHAM | 96 | 9 | 9.4 | 43816 | 219 | 21 |
| QLD | 31802 | TOWNSVILLE | 785 | 55 | 7.0 | 188107 | 417 | 29 |
| QLD | 31901 | BUNDABERG | 60 | 3 | 5.0 | 88649 | 68 | 3 |
| QLD | 31902 | BURNETT | 31 | 3 | 9.7 | 49645 | 62 | 6 |
| QLD | 31903 | GYMPIE - COOLOOLA | 24 | 0 | 0.0 | 48973 | 49 | 0 |
| QLD | 31904 | HERVEY BAY | 49 | 1 | 2.0 | 56354 | 87 | 2 |
| QLD | 31905 | MARYBOROUGH | 26 | 0 | 0.0 | 44857 | 58 | 0 |
| TAS | 60101 | BRIGHTON | 48 | 0 | 0.0 | 16809 | 286 | 0 |
| TAS | 60102 | HOBART - NORTH EAST | 306 | 12 | 3.9 | 52080 | 588 | 23 |
| TAS | 60103 | HOBART - NORTH WEST | 955 | 52 | 5.4 | 52898 | 1805 | 98 |
| TAS | 60104 | HOBART - SOUTH AND WEST | 38 | 2 | 5.3 | 32748 | 116 | 6 |
| TAS | 60105 | HOBART INNER | 234 | 8 | 3.4 | 51377 | 455 | 16 |
| TAS | 60106 | SORELL - DODGES FERRY | 24 | 0 | 0.0 | 15641 | 153 | 0 |
| TAS | 60201 | LAUNCESTON | 1156 | 39 | 3.4 | 82408 | 1403 | 47 |
| TAS | 60202 | MEANDER VALLEY - WEST TAMAR | 26 | 1 | 3.8 | 22708 | 114 | 4 |
| TAS | 60203 | NORTH EAST | 28 | 0 | 0.0 | 37785 | 74 | 0 |
| TAS | 60301 | CENTRAL HIGHLANDS (TAS.) | 7 | 0 | 0.0 | 11503 | 61 | 0 |
| TAS | 60302 | HUON - BRUNY ISLAND | 18 | 1 | 5.6 | 19240 | 94 | 5 |
| TAS | 60303 | SOUTH EAST COAST | 7 | 0 | 0.0 | 6797 | 103 | 0 |
| TAS | 60401 | BURNIE - ULVERSTONE | 39 | 1 | 2.6 | 49038 | 80 | 2 |
| TAS | 60402 | DEVONPORT | 40 | 1 | 2.5 | 45004 | 89 | 2 |
| TAS | 60403 | WEST COAST | 13 | 0 | 0.0 | 18004 | 72 | 0 |
| VIC | 20101 | BALLARAT | 110 | 5 | 4.6 | 102140 | 108 | 5 |
| VIC | 20102 | CRESWICK - DAYLESFORD - BALLAN | 18 | 0 | 0.0 | 28134 | 64 | 0 |
| VIC | 20103 | MARYBOROUGH - PYRENEES | 26 | 1 | 3.8 | 24953 | 104 | 4 |
| VIC | 20201 | BENDIGO | 233 | 16 | 6.9 | 92439 | 252 | 17 |
| VIC | 20202 | HEATHCOTE - CASTLEMAINE - KYNETON | 100 | 4 | 4.0 | 46327 | 216 | 9 |
| VIC | 20203 | LODDON - ELMORE | 18 | 0 | 0.0 | 11402 | 158 | 0 |
| VIC | 20301 | BARWON - WEST | 19 | 0 | 0.0 | 18357 | 104 | 0 |
| VIC | 20302 | GEELONG | 1155 | 39 | 3.4 | 186033 | 621 | 21 |
| VIC | 20303 | SURF COAST - BELLARINE PENINSULA | 94 | 3 | 3.2 | 68600 | 137 | 4 |
| VIC | 20401 | UPPER GOULBURN VALLEY | 73 | 3 | 4.1 | 52875 | 138 | 6 |
| VIC | 20402 | WANGARATTA - BENALLA | 34 | 0 | 0.0 | 45732 | 74 | 0 |
| VIC | 20403 | WODONGA - ALPINE | 82 | 11 | 13.4 | 69157 | 119 | 16 |
| VIC | 20501 | BAW BAW | 27 | 1 | 3.7 | 46932 | 58 | 2 |
| VIC | 20502 | GIPPSLAND - EAST | 60 | 4 | 6.7 | 44491 | 135 | 9 |
| VIC | 20503 | GIPPSLAND - SOUTH WEST | 54 | 3 | 5.6 | 60709 | 89 | 5 |
| VIC | 20504 | LATROBE VALLEY | 46 | 2 | 4.3 | 74058 | 62 | 3 |
| VIC | 20505 | WELLINGTON | 27 | 1 | 3.7 | 42849 | 63 | 2 |
| VIC | 20601 | BRUNSWICK - COBURG | 372 | 19 | 5.1 | 88073 | 422 | 22 |
| VIC | 20602 | DAREBIN - SOUTH | 199 | 11 | 5.5 | 53497 | 372 | 21 |
| VIC | 20603 | ESSENDON | 324 | 20 | 6.2 | 67393 | 481 | 30 |
| VIC | 20604 | MELBOURNE CITY | 1647 | 107 | 6.5 | 127445 | 1292 | 84 |
| VIC | 20605 | PORT PHILLIP | 289 | 17 | 5.9 | 104313 | 277 | 16 |
| VIC | 20606 | STONNINGTON - WEST | 206 | 7 | 3.4 | 63218 | 326 | 11 |
| VIC | 20607 | YARRA | 557 | 34 | 6.1 | 87872 | 634 | 39 |
| VIC | 20701 | BOROONDARA | 448 | 22 | 4.9 | 172731 | 259 | 13 |
| VIC | 20702 | MANNINGHAM - WEST | 271 | 8 | 3.0 | 92864 | 292 | 9 |
| VIC | 20703 | WHITEHORSE - WEST | 228 | 10 | 4.4 | 103401 | 220 | 10 |
| VIC | 20801 | BAYSIDE | 165 | 8 | 4.8 | 99840 | 165 | 8 |
| VIC | 20802 | GLEN EIRA | 277 | 18 | 6.5 | 151484 | 183 | 12 |
| VIC | 20803 | KINGSTON | 235 | 12 | 5.1 | 118590 | 198 | 10 |
| VIC | 20804 | STONNINGTON - EAST | 87 | 1 | 1.1 | 42746 | 204 | 2 |
| VIC | 20901 | BANYULE | 461 | 27 | 5.9 | 125308 | 368 | 22 |
| VIC | 20902 | DAREBIN - NORTH | 404 | 31 | 7.7 | 96276 | 420 | 32 |
| VIC | 20903 | NILLUMBIK - KINGLAKE | 128 | 9 | 7.0 | 67343 | 190 | 13 |
| VIC | 20904 | WHITTLESEA - WALLAN | 692 | 30 | 4.3 | 203102 | 341 | 15 |
| VIC | 21001 | KEILOR | 214 | 8 | 3.7 | 59407 | 360 | 13 |
| VIC | 21002 | MACEDON RANGES | 44 | 1 | 2.3 | 29261 | 150 | 3 |
| VIC | 21003 | MORELAND - NORTH | 356 | 21 | 5.9 | 75416 | 472 | 28 |
| VIC | 21004 | SUNBURY | 66 | 3 | 4.6 | 39479 | 167 | 8 |
| VIC | 21005 | TULLAMARINE - BROADMEADOWS | 807 | 55 | 6.8 | 154835 | 521 | 36 |
| VIC | 21101 | KNOX | 258 | 14 | 5.4 | 157693 | 164 | 9 |
| VIC | 21102 | MANNINGHAM - EAST | 57 | 3 | 5.3 | 26970 | 211 | 11 |
| VIC | 21103 | MAROONDAH | 1147 | 124 | 10.8 | 111051 | 1033 | 112 |
| VIC | 21104 | WHITEHORSE - EAST | 161 | 10 | 6.2 | 61820 | 260 | 16 |
| VIC | 21105 | YARRA RANGES | 514 | 50 | 9.7 | 151436 | 339 | 33 |
| VIC | 21201 | CARDINIA | 93 | 11 | 11.8 | 88761 | 105 | 12 |
| VIC | 21202 | CASEY - NORTH | 728 | 53 | 7.3 | 133622 | 545 | 40 |
| VIC | 21203 | CASEY - SOUTH | 373 | 20 | 5.4 | 155881 | 239 | 13 |
| VIC | 21204 | DANDENONG | 3034 | 251 | 8.3 | 188250 | 1612 | 133 |
| VIC | 21205 | MONASH | 342 | 20 | 5.8 | 178534 | 192 | 11 |
| VIC | 21301 | BRIMBANK | 1429 | 86 | 6.0 | 189882 | 753 | 45 |
| VIC | 21302 | HOBSONS BAY | 217 | 12 | 5.5 | 85744 | 253 | 14 |
| VIC | 21303 | MARIBYRNONG | 544 | 31 | 5.7 | 82230 | 662 | 38 |
| VIC | 21304 | MELTON - BACCHUS MARSH | 343 | 14 | 4.1 | 146977 | 233 | 10 |
| VIC | 21305 | WYNDHAM | 1836 | 175 | 9.5 | 207204 | 886 | 84 |
| VIC | 21401 | FRANKSTON | 150 | 5 | 3.3 | 136004 | 110 | 4 |
| VIC | 21402 | MORNINGTON PENINSULA | 296 | 17 | 5.7 | 156512 | 189 | 11 |
| VIC | 21501 | GRAMPIANS | 98 | 6 | 6.1 | 59730 | 164 | 10 |
| VIC | 21502 | MILDURA | 663 | 26 | 3.9 | 53636 | 1236 | 48 |
| VIC | 21503 | MURRAY RIVER - SWAN HILL | 55 | 6 | 10.9 | 37822 | 145 | 16 |
| VIC | 21601 | CAMPASPE | 39 | 2 | 5.1 | 37482 | 104 | 5 |
| VIC | 21602 | MOIRA | 27 | 2 | 7.4 | 29091 | 93 | 7 |
| VIC | 21603 | SHEPPARTON | 281 | 7 | 2.5 | 63664 | 441 | 11 |
| VIC | 21701 | GLENELG - SOUTHERN GRAMPIANS | 24 | 0 | 0.0 | 36106 | 66 | 0 |
| VIC | 21702 | WARRNAMBOOL - OTWAY RANGES | 94 | 4 | 4.3 | 87648 | 107 | 5 |
| WA | 50101 | AUGUSTA - MARGARET RIVER - BUSSELTON | 153 | 3 | 2.0 | 49552 | 309 | 6 |
| WA | 50102 | BUNBURY | 155 | 7 | 4.5 | 102917 | 151 | 7 |
| WA | 50103 | MANJIMUP | 30 | 0 | 0.0 | 22964 | 131 | 0 |
| WA | 50201 | MANDURAH | 89 | 3 | 3.4 | 96075 | 93 | 3 |
| WA | 50301 | COTTESLOE - CLAREMONT | 188 | 6 | 3.2 | 71045 | 265 | 8 |
| WA | 50302 | PERTH CITY | 339 | 8 | 2.4 | 105418 | 322 | 8 |
| WA | 50401 | BAYSWATER - BASSENDEAN | 406 | 12 | 3.0 | 83881 | 484 | 14 |
| WA | 50402 | MUNDARING | 57 | 8 | 14.0 | 43462 | 131 | 18 |
| WA | 50403 | SWAN | 382 | 26 | 6.8 | 123702 | 309 | 21 |
| WA | 50501 | JOONDALUP | 203 | 6 | 3.0 | 163325 | 124 | 4 |
| WA | 50502 | STIRLING | 1975 | 52 | 2.6 | 199687 | 989 | 26 |
| WA | 50503 | WANNEROO | 961 | 36 | 3.8 | 182025 | 528 | 20 |
| WA | 50601 | ARMADALE | 234 | 9 | 3.8 | 75638 | 309 | 12 |
| WA | 50602 | BELMONT - VICTORIA PARK | 335 | 9 | 2.7 | 72564 | 462 | 12 |
| WA | 50603 | CANNING | 383 | 16 | 4.2 | 97011 | 395 | 16 |
| WA | 50604 | GOSNELLS | 1073 | 23 | 2.1 | 120015 | 894 | 19 |
| WA | 50605 | KALAMUNDA | 172 | 10 | 5.8 | 58781 | 293 | 17 |
| WA | 50606 | SERPENTINE - JARRAHDALE | 34 | 0 | 0.0 | 23851 | 143 | 0 |
| WA | 50607 | SOUTH PERTH | 91 | 3 | 3.3 | 44190 | 206 | 7 |
| WA | 50701 | COCKBURN | 132 | 10 | 7.6 | 101863 | 130 | 10 |
| WA | 50702 | FREMANTLE | 97 | 4 | 4.1 | 38127 | 254 | 10 |
| WA | 50703 | KWINANA | 171 | 6 | 3.5 | 36410 | 470 | 16 |
| WA | 50704 | MELVILLE | 182 | 5 | 2.8 | 106350 | 171 | 5 |
| WA | 50705 | ROCKINGHAM | 122 | 4 | 3.3 | 122351 | 100 | 3 |
| WA | 50801 | ESPERANCE | 33 | 6 | 18.2 | 16495 | 200 | 36 |
| WA | 50802 | GASCOYNE | 48 | 3 | 6.3 | 9834 | 488 | 31 |
| WA | 50803 | GOLDFIELDS | 186 | 13 | 7.0 | 42811 | 434 | 30 |
| WA | 50804 | KIMBERLEY | 1674 | 374 | 22.3 | 37542 | 4459 | 996 |
| WA | 50805 | MID WEST | 99 | 10 | 10.1 | 56302 | 176 | 18 |
| WA | 50806 | PILBARA | 459 | 42 | 9.1 | 63488 | 723 | 66 |
| WA | 50901 | ALBANY | 127 | 3 | 2.4 | 59695 | 213 | 5 |
| WA | 50902 | WHEAT BELT - NORTH | 86 | 5 | 5.8 | 56798 | 151 | 9 |
| WA | 50903 | WHEAT BELT - SOUTH | 16 | 1 | 6.3 | 21177 | 76 | 5 |
| SA | 40101 | ADELAIDE CITY | 29 | 3 | 10.3 | 22588 | 128 | 13 |
| SA | 40102 | ADELAIDE HILLS | 8 | 0 | 0.0 | 71727 | 11 | 0 |
| SA | 40103 | BURNSIDE | 12 | 0 | 0.0 | 44714 | 27 | 0 |
| SA | 40104 | CAMPBELLTOWN (SA) | 14 | 1 | 7.1 | 51047 | 27 | 2 |
| SA | 40105 | NORWOOD - PAYNEHAM - ST PETERS | 12 | 2 | 16.7 | 36363 | 33 | 6 |
| SA | 40106 | PROSPECT - WALKERVILLE | 4 | 0 | 0.0 | 28559 | 14 | 0 |
| SA | 40107 | UNLEY | 5 | 1 | 20.0 | 38705 | 13 | 3 |
| SA | 40201 | GAWLER - TWO WELLS | 6 | 0 | 0.0 | 33818 | 18 | 0 |
| SA | 40202 | PLAYFORD | 78 | 5 | 6.4 | 87827 | 89 | 6 |
| SA | 40203 | PORT ADELAIDE - EAST | 59 | 0 | 0.0 | 66797 | 88 | 0 |
| SA | 40204 | SALISBURY | 226 | 18 | 8.0 | 135895 | 166 | 13 |
| SA | 40205 | TEA TREE GULLY | 22 | 3 | 13.6 | 94661 | 23 | 3 |
| SA | 40301 | HOLDFAST BAY | 12 | 1 | 8.3 | 34695 | 35 | 3 |
| SA | 40302 | MARION | 17 | 2 | 11.8 | 90116 | 19 | 2 |
| SA | 40303 | MITCHAM | 16 | 0 | 0.0 | 64642 | 25 | 0 |
| SA | 40304 | ONKAPARINGA | 33 | 5 | 15.1 | 168592 | 20 | 3 |
| SA | 40401 | CHARLES STURT | 36 | 5 | 13.9 | 109599 | 33 | 5 |
| SA | 40402 | PORT ADELAIDE - WEST | 27 | 0 | 0.0 | 59313 | 46 | 0 |
| SA | 40403 | WEST TORRENS | 9 | 0 | 0.0 | 61761 | 15 | 0 |
| SA | 40501 | BAROSSA | 2 | 0 | 0.0 | 35321 | 6 | 0 |
| SA | 40502 | LOWER NORTH | 5 | 1 | 20.0 | 22828 | 22 | 4 |
| SA | 40503 | MID NORTH | 2 | 0 | 0.0 | 27886 | 7 | 0 |
| SA | 40504 | YORKE PENINSULA | 25 | 4 | 16.0 | 25257 | 99 | 16 |
| SA | 40601 | EYRE PENINSULA AND SOUTH WEST | 1 | 0 | 0.0 | 58306 | 2 | 0 |
| SA | 40602 | OUTBACK - NORTH AND EAST | 7 | 0 | 0.0 | 28288 | 25 | 0 |
| SA | 40701 | FLEURIEU - KANGAROO ISLAND | 7 | 1 | 14.3 | 50006 | 14 | 2 |
| SA | 40702 | LIMESTONE COAST | 31 | 5 | 16.1 | 65926 | 47 | 8 |
| SA | 40703 | MURRAY AND MALLEE | 3 | 0 | 0.0 | 70495 | 4 | 0 |
| Total |  |  | 81146 | 6866 | 8.5 | 23465538 | 346 | 29 |
