# Supplementary material for: Seropositivity and geographical distribution of Strongyloides stercoralis in Australia: A study of pathology laboratory data from 2012–2016
Source: PLoS Negl Trop Dis. 2021 Mar 9;15(3):e0009160. doi: 10.1371/journal.pntd.0009160 (PMC7978363; doi:10.1371/journal.pntd.0009160)
Supplement: S5 Table — The number of people were calculated separately for each year. 1The number of people were calculated separately for each year so a person who was tested in more than one year appears more than once in the data. ACT = Australian Capital Territory; NSW = New South Wales; NT = Northern Territory; QLD = Queensland; SA = South Australia; TAS = Tasmania; VIC = Victoria;, WA = Western Australia. (DOCX) [file pntd.0009160.s009.docx]

| **State /Territory** | **Year^1^** | **No. Tested** | **No. Positive** | **% Positive** | **Population** | **No. Tested /100000** | **No. Positive /100000** |
| --- | --- | --- | --- | --- | --- | --- | --- |
| ACT | 2012 | 343 | 21 | 6.1 | 376539 | 91 | 6 |
| ACT | 2013 | 565 | 27 | 4.8 | 383257 | 147 | 7 |
| ACT | 2014 | 476 | 29 | 6.1 | 388799 | 122 | 7 |
| ACT | 2015 | 496 | 24 | 4.8 | 395813 | 125 | 6 |
| ACT | 2016 | 619 | 40 | 6.5 | 403104 | 154 | 10 |
| NSW | 2012 | 2027 | 285 | 14.1 | 7304225 | 28 | 4 |
| NSW | 2013 | 4585 | 367 | 8.0 | 7404008 | 62 | 5 |
| NSW | 2014 | 4974 | 448 | 9.0 | 7508311 | 66 | 6 |
| NSW | 2015 | 3967 | 386 | 9.7 | 7616124 | 52 | 5 |
| NSW | 2016 | 5942 | 535 | 9.0 | 7732846 | 77 | 7 |
| QLD | 2012 | 2354 | 258 | 11.0 | 4568687 | 52 | 6 |
| QLD | 2013 | 3238 | 323 | 10.0 | 4652824 | 70 | 7 |
| QLD | 2014 | 3018 | 360 | 11.9 | 4719653 | 64 | 8 |
| QLD | 2015 | 2705 | 331 | 12.2 | 4777692 | 57 | 7 |
| QLD | 2016 | 3182 | 354 | 11.1 | 4845152 | 66 | 7 |
| TAS | 2012 | 414 | 35 | 8.4 | 511724 | 81 | 7 |
| TAS | 2013 | 738 | 33 | 4.5 | 512231 | 144 | 6 |
| TAS | 2014 | 623 | 20 | 3.2 | 513621 | 121 | 4 |
| TAS | 2015 | 463 | 13 | 2.8 | 515117 | 90 | 3 |
| TAS | 2016 | 749 | 27 | 3.6 | 517514 | 145 | 5 |
| VIC | 2012 | 3513 | 347 | 9.9 | 5651091 | 62 | 6 |
| VIC | 2013 | 5787 | 447 | 7.7 | 5772669 | 100 | 8 |
| VIC | 2014 | 5442 | 407 | 7.5 | 5894917 | 92 | 7 |
| VIC | 2015 | 4466 | 249 | 5.6 | 6022322 | 74 | 4 |
| VIC | 2016 | 5006 | 226 | 4.5 | 6173172 | 81 | 4 |
| WA | 2012 | 2993 | 310 | 10.4 | 2425507 | 123 | 13 |
| WA | 2013 | 2394 | 168 | 7.0 | 2486944 | 96 | 7 |
| WA | 2014 | 1919 | 92 | 4.8 | 2517608 | 76 | 4 |
| WA | 2015 | 1886 | 97 | 5.1 | 2540672 | 74 | 4 |
| WA | 2016 | 2207 | 87 | 3.9 | 2555978 | 86 | 3 |
| SA | 2012 | 80 | 7 | 8.8 | 1656725 | 5 | 0 |
| SA | 2013 | 83 | 9 | 10.8 | 1671488 | 5 | 1 |
| SA | 2014 | 69 | 10 | 14.5 | 1686945 | 4 | 1 |
| SA | 2015 | 196 | 14 | 7.1 | 1700668 | 12 | 1 |
| SA | 2016 | 292 | 26 | 8.9 | 1712843 | 17 | 2 |
| NT | 2012 | 3102 | 614 | 19.8 | 235915 | 1315 | 260 |
| NT | 2013 | 2197 | 249 | 11.3 | 241722 | 909 | 103 |
| NT | 2014 | 1906 | 197 | 10.3 | 242894 | 785 | 81 |
| NT | 2015 | 685 | 69 | 10.1 | 244692 | 280 | 28 |
| NT | 2016 | 169 | 6 | 3.5 | 245678 | 69 | 2 |
| Australia | 2012 | 14826 | 1877 | 12.7 | 22730413 | 65 | 8 |
| Australia | 2013 | 19587 | 1623 | 8.3 | 23125143 | 85 | 7 |
| Australia | 2014 | 18427 | 1563 | 8.5 | 23472748 | 79 | 7 |
| Australia | 2015 | 14864 | 1183 | 8.0 | 23813100 | 62 | 5 |
| Australia | 2016 | 18166 | 1301 | 7.2 | 24186287 | 75 | 5 |
