# Supplementary material for: Seropositivity and geographical distribution of Strongyloides stercoralis in Australia: A study of pathology laboratory data from 2012–2016
Source: PLoS Negl Trop Dis. 2021 Mar 9;15(3):e0009160. doi: 10.1371/journal.pntd.0009160 (PMC7978363; doi:10.1371/journal.pntd.0009160)
Supplement: S6 Table — F = female, M = male, U = unknown. ACT = Australian Capital Territory; NSW = New South Wales; NT = Northern Territory; QLD = Queensland; SA = South Australia; TAS = Tasmania; VIC = Victoria; WA = Western Australia. (DOCX) [file pntd.0009160.s010.docx]

| **State /Territory** | **Sex** | **No. Tested** | **No. Positive** | **% Positive** | **Annualized Average Population** | **No. Tested /100,000** | **No. Positive /100,000** |
| --- | --- | --- | --- | --- | --- | --- | --- |
| ACT | F | 824 | 48 | 5.8 | 196212 | 420 | 24 |
| ACT | M | 1317 | 69 | 5.2 | 193291 | 681 | 36 |
| ACT | U | 4 | 0 | 0.0 |  |  |  |
| NSW | F | 9971 | 793 | 7.9 | 3786002 | 263 | 21 |
| NSW | M | 10713 | 1011 | 9.4 | 3727100 | 287 | 27 |
| NSW | U | 33 | 9 | 27.3 |  |  |  |
| QLD | F | 6333 | 617 | 9.7 | 2367787 | 267 | 26 |
| QLD | M | 7095 | 812 | 11.4 | 2345014 | 303 | 35 |
| QLD | U | 30 | 2 | 6.7 |  |  |  |
| TAS | F | 1485 | 56 | 3.8 | 258823 | 574 | 22 |
| TAS | M | 1451 | 61 | 4.2 | 255218 | 569 | 24 |
| TAS | U | 3 | 0 | 0.0 |  |  |  |
| VIC | F | 10075 | 640 | 6.3 | 2983581 | 338 | 21 |
| VIC | M | 13296 | 871 | 6.6 | 2919253 | 455 | 30 |
| VIC | U | 85 | 10 | 11.8 |  |  |  |
| WA | F | 4952 | 363 | 7.3 | 1244643 | 398 | 29 |
| WA | M | 5739 | 360 | 6.3 | 1260699 | 455 | 29 |
| WA | U | 1 | 0 | 0.0 |  |  |  |
| SA | F | 332 | 19 | 5.7 | 851308 | 39 | 2 |
| SA | M | 375 | 38 | 10.1 | 834425 | 45 | 5 |
| SA | U | 1 | 0 | 0.0 |  |  |  |
| NT | F | 3504 | 517 | 14.8 | 115573 | 3032 | 447 |
| NT | M | 3465 | 565 | 16.3 | 126607 | 2737 | 446 |
| NT | U | 62 | 5 | 8.1 |  |  |  |
| Australia | F | 37476 | 3053 | 8.1 | 11803929 | 317 | 26 |
| Australia | M | 43451 | 3787 | 8.7 | 11661609 | 373 | 33 |
| Australia | U | 219 | 26 | 11.9 |  |  |  |
| Total |  | 81146 | 6866 | 8.5 | 23465538 | 346 | 29 |
