# Supplementary material for: Seropositivity and geographical distribution of Strongyloides stercoralis in Australia: A study of pathology laboratory data from 2012–2016
Source: PLoS Negl Trop Dis. 2021 Mar 9;15(3):e0009160. doi: 10.1371/journal.pntd.0009160 (PMC7978363; doi:10.1371/journal.pntd.0009160)
Supplement: S7 Table — ACT = Australian Capital Territory; NSW = New South Wales; NT = Northern Territory; QLD = Queensland; SA = South Australia; TAS = Tasmania; VIC = Victoria; WA = Western Australia. (DOCX) [file pntd.0009160.s011.docx]

| **State /Territory** | **Age Group (years)** | **No. Tested** | **No. Positive** | **% Positive** | **Population** | **No. tested /100000** | **No. Positive /100000** |
| --- | --- | --- | --- | --- | --- | --- | --- |
| ACT | 0-4 | 63 | 1 | 1.6 | 26786 | 235 | 4 |
| ACT | 5-14 | 193 | 2 | 1.0 | 45561 | 424 | 4 |
| ACT | 15-24 | 187 | 9 | 4.8 | 57597 | 325 | 16 |
| ACT | 25-34 | 471 | 23 | 4.9 | 66893 | 704 | 34 |
| ACT | 35-44 | 556 | 28 | 5.0 | 56955 | 976 | 49 |
| ACT | 45-54 | 403 | 25 | 6.2 | 49760 | 810 | 50 |
| ACT | 55-64 | 182 | 20 | 11.0 | 40516 | 449 | 49 |
| ACT | 65-74 | 68 | 7 | 10.3 | 26762 | 254 | 26 |
| ACT | >=75 | 20 | 2 | 10.0 | 18673 | 107 | 11 |
| ACT | Unknown | 2 | 0 | 0.0 |  |  |  |
| NSW | 0-4 | 752 | 10 | 1.3 | 491689 | 153 | 2 |
| NSW | 5-14 | 3031 | 74 | 2.4 | 924839 | 328 | 8 |
| NSW | 15-24 | 3032 | 121 | 4.0 | 979124 | 310 | 12 |
| NSW | 25-34 | 3236 | 242 | 7.5 | 1089680 | 297 | 22 |
| NSW | 35-44 | 3100 | 288 | 9.3 | 1023402 | 303 | 28 |
| NSW | 45-54 | 2656 | 371 | 14.0 | 984915 | 270 | 38 |
| NSW | 55-64 | 2292 | 329 | 14.4 | 867844 | 264 | 38 |
| NSW | 65-74 | 1610 | 226 | 14.0 | 635654 | 253 | 36 |
| NSW | >=75 | 963 | 143 | 14.9 | 515956 | 187 | 28 |
| NSW | Unknown | 45 | 9 | 20.0 |  |  |  |
| QLD | 0-4 | 322 | 5 | 1.5 | 316400 | 102 | 2 |
| QLD | 5-14 | 1331 | 50 | 3.8 | 617162 | 216 | 8 |
| QLD | 15-24 | 2536 | 310 | 12.2 | 642859 | 394 | 48 |
| QLD | 25-34 | 2399 | 256 | 10.7 | 669617 | 358 | 38 |
| QLD | 35-44 | 2021 | 225 | 11.1 | 646425 | 313 | 35 |
| QLD | 45-54 | 1663 | 208 | 12.5 | 626189 | 266 | 33 |
| QLD | 55-64 | 1356 | 180 | 13.3 | 534337 | 254 | 34 |
| QLD | 65-74 | 1049 | 125 | 11.9 | 386498 | 271 | 32 |
| QLD | >=75 | 774 | 70 | 9.0 | 273314 | 283 | 26 |
| QLD | Unknown | 7 | 2 | 28.6 |  |  |  |
| TAS | 0-4 | 214 | 5 | 2.3 | 30812 | 695 | 16 |
| TAS | 5-14 | 629 | 21 | 3.3 | 63191 | 995 | 33 |
| TAS | 15-24 | 562 | 15 | 2.7 | 64265 | 875 | 23 |
| TAS | 25-34 | 454 | 18 | 4.0 | 60055 | 756 | 30 |
| TAS | 35-44 | 425 | 27 | 6.3 | 63002 | 675 | 43 |
| TAS | 45-54 | 264 | 11 | 4.2 | 71313 | 370 | 15 |
| TAS | 55-64 | 186 | 9 | 4.8 | 70029 | 266 | 13 |
| TAS | 65-74 | 131 | 7 | 5.3 | 52613 | 249 | 13 |
| TAS | >=75 | 73 | 4 | 5.5 | 38762 | 188 | 10 |
| TAS | Unknown | 1 | 0 | 0.0 |  |  |  |
| VIC | 0-4 | 671 | 26 | 3.9 | 381396 | 176 | 7 |
| VIC | 5-14 | 2210 | 84 | 3.8 | 704070 | 314 | 12 |
| VIC | 15-24 | 3732 | 223 | 6.0 | 795357 | 469 | 28 |
| VIC | 25-34 | 5235 | 343 | 6.6 | 900843 | 581 | 38 |
| VIC | 35-44 | 3692 | 286 | 7.8 | 819062 | 451 | 35 |
| VIC | 45-54 | 2745 | 194 | 7.1 | 773646 | 355 | 25 |
| VIC | 55-64 | 2431 | 158 | 6.5 | 659847 | 368 | 24 |
| VIC | 65-74 | 1599 | 103 | 6.4 | 477232 | 335 | 22 |
| VIC | >=75 | 1122 | 98 | 8.7 | 391380 | 287 | 25 |
| VIC | Unknown | 19 | 6 | 31.6 |  |  |  |
| WA | 0-4 | 500 | 16 | 3.2 | 169216 | 295 | 9 |
| WA | 5-14 | 1380 | 52 | 3.8 | 314461 | 439 | 17 |
| WA | 15-24 | 1737 | 136 | 7.8 | 335049 | 518 | 41 |
| WA | 25-34 | 2105 | 129 | 6.1 | 397054 | 530 | 32 |
| WA | 35-44 | 1764 | 131 | 7.4 | 353701 | 499 | 37 |
| WA | 45-54 | 1320 | 116 | 8.8 | 335410 | 394 | 35 |
| WA | 55-64 | 972 | 79 | 8.1 | 277512 | 350 | 28 |
| WA | 65-74 | 576 | 41 | 7.1 | 185844 | 310 | 22 |
| WA | >=75 | 338 | 23 | 6.8 | 137095 | 247 | 17 |
| SA | 0-4 | 9 | 0 | 0.0 | 101656 | 9 | 0 |
| SA | 5-14 | 96 | 4 | 4.2 | 197645 | 49 | 2 |
| SA | 15-24 | 147 | 5 | 3.4 | 220247 | 67 | 2 |
| SA | 25-34 | 110 | 8 | 7.3 | 225351 | 49 | 4 |
| SA | 35-44 | 116 | 9 | 7.8 | 217114 | 53 | 4 |
| SA | 45-54 | 82 | 7 | 8.5 | 228701 | 36 | 3 |
| SA | 55-64 | 72 | 11 | 15.3 | 207999 | 35 | 5 |
| SA | 65-74 | 53 | 9 | 17.0 | 155569 | 34 | 6 |
| SA | >=75 | 21 | 4 | 19.0 | 131451 | 16 | 3 |
| SA | Unknown | 2 | 0 | 0.0 |  |  |  |
| NT | 0-4 | 257 | 21 | 8.2 | 19195 | 1339 | 109 |
| NT | 5-14 | 339 | 42 | 12.4 | 34138 | 993 | 123 |
| NT | 15-24 | 599 | 148 | 24.7 | 34918 | 1715 | 424 |
| NT | 25-34 | 969 | 222 | 22.9 | 46021 | 2106 | 482 |
| NT | 35-44 | 1219 | 212 | 17.4 | 36754 | 3317 | 577 |
| NT | 45-54 | 1486 | 228 | 15.3 | 32101 | 4629 | 710 |
| NT | 55-64 | 1230 | 145 | 11.8 | 23609 | 5210 | 614 |
| NT | 65-74 | 629 | 45 | 7.2 | 11183 | 5624 | 402 |
| NT | >=75 | 303 | 24 | 7.9 | 4260 | 7113 | 563 |
| NT | Unknown | 0 | 0 | 0.0 |  |  |  |
| Australia | 0-4 | 2788 | 84 | 3.0 | 1537150 | 181 | 5 |
| Australia | 5-14 | 9209 | 329 | 3.6 | 2901067 | 317 | 11 |
| Australia | 15-24 | 12532 | 967 | 7.7 | 3129417 | 400 | 31 |
| Australia | 25-34 | 14979 | 1241 | 8.3 | 3455515 | 433 | 36 |
| Australia | 35-44 | 12893 | 1206 | 9.4 | 3216414 | 401 | 37 |
| Australia | 45-54 | 10619 | 1160 | 10.9 | 3102036 | 342 | 37 |
| Australia | 55-64 | 8721 | 931 | 10.7 | 2681694 | 325 | 35 |
| Australia | 65-74 | 5715 | 563 | 9.9 | 1931354 | 296 | 29 |
| Australia | ≥75 | 3614 | 368 | 10.2 | 1510891 | 239 | 24 |
| Australia | Unknown | 76 | 17 | 22.4 |  |  |  |
| Total |  | 81146 | 6866 | 8.5 | 23465538 | 346 | 29 |
